# Differential Association of the Conserved SUMO Ligase Zip3 with Meiotic Double-Strand Break Sites Reveals Regional Variations in the Outcome of Meiotic Recombination

**DOI:** 10.1371/journal.pgen.1003416

**Published:** 2013-04-04

**Authors:** Maria-Elisabetta Serrentino, Emmanuel Chaplais, Vérane Sommermeyer, Valérie Borde

**Affiliations:** 1 Institut Curie, Centre de Recherche, Paris, France; 2 CNRS, UMR 218, Paris, France; The University of North Carolina at Chapel Hill, United States of America

## Abstract

During the first meiotic prophase, programmed DNA double-strand breaks (DSBs) are distributed non randomly at hotspots along chromosomes, to initiate recombination. In all organisms, more DSBs are formed than crossovers (CO), the repair product that creates a physical link between homologs and allows their correct segregation. It is not known whether all DSB hotspots are also CO hotspots or if the CO/DSB ratio varies with the chromosomal location. Here, we investigated the variations in the CO/DSB ratio by mapping genome-wide the binding sites of the Zip3 protein during budding yeast meiosis. We show that Zip3 associates with DSB sites that are engaged in repair by CO, and Zip3 enrichment at DSBs reflects the DSB tendency to be repaired by CO. Moreover, the relative amount of Zip3 per DSB varies with the chromosomal location, and specific chromosomal features are associated with high or low Zip3 per DSB. This work shows that DSB hotspots are not necessarily CO hotspots and suggests that different categories of DSB sites may fulfill different functions.

## Author Summary

For sexual reproduction, meiosis is an essential step ensuring the formation of haploid gametes from diploid precursors of the germline. This reduction in the genome's content is achieved through a specialized type of division, where a single round of DNA replication is followed by two successive rounds of chromosome segregation. The first round separates the homologs. For this to faithfully occur, homologous chromosomes pair with each other and experience recombination, catalyzed by the formation of programmed double-strand breaks (DSBs). Upon their repair, a subset of DSBs will generate crossovers, which result from an intermediate that creates a physical link between homologs and allows their correct segregation by the meiotic spindle. DSBs, as well as crossovers, do not occur randomly along chromosomes but at preferential places called hotspots. To ask if all DSB hotspots also give rise to high crossover frequency, we have systematically compared the map of DSBs with that of a protein, Zip3, which we show preferentially binds to DSB sites that are being repaired with a crossover. We discovered that several DSB hotspots rarely produce crossovers, meaning that the decision to repair a DSB with a crossover can be influenced by specific chromosomal features.

## Introduction

During meiosis, the programmed formation of DNA double-strand breaks (DSBs) and their repair by homologous recombination ensures that crossovers (CO) occur between homologous chromosomes. COs promote the accurate segregation of homologs at the first meiotic division, thus avoiding aneuploidy, which is a common cause of birth defects and congenital diseases. In all species, two to 30 times more DSBs are formed than COs, indicating that only a subset of all DSBs formed in a cell are repaired through a pathway that will give rise to a CO. The remaining DSBs are repaired by other homologous recombination pathways, such as the synthesis dependent strand annealing (SDSA) mechanism, symmetrical Holliday junction resolution or Holliday junction dissolution [1], that result in non-crossovers (NCOs). In addition, a substantial fraction of meiotic DSBs is also repaired by homologous recombination using the sister chromatid as template, which is not productive for chiasmata and homolog segregation [2]. The repair pathway choice has thus to be tightly controlled to ensure the required number of COs per homolog pair.

DSBs and COs tend to occur more frequently at preferred sites, or hotspots. It is not known whether DSB hotspots are also CO hotspots, or whether DSB repair is modulated by DSB localization on a chromosome. This question could be answered by comparing a high resolution genome-wide map of CO frequencies to the existing high resolution maps of DSBs, for instance in budding yeast (e.g., [3], [4]). Nevertheless, several studies have suggested that the relative contribution of each DSB repair pathway may vary from site to site along the genome. For instance, using a small number of yeast meioses, Mancera et al noted that some sites gave rise to more COs and others to more NCOs per total recombination events [5]. Using a similar approach, Fung and colleagues showed that close to centromeres, COs and NCOs are strongly repressed although DSB activity was reported in these regions, suggesting that DSBs in centromere-proximal chromosomal regions are preferentially repaired by sister chromatid recombination [6], [7]. Analyses of human sperm recombination frequencies revealed that the CO/NCO ratio varied 30 times in the sites under study [8], [9], [10]. Finally, in the fission yeast *Schizosaccharomyces pombe*, strong discrepancies were found between the DSB map and the CO frequencies [11]. Thus, it is worth investigating if the map of meiotic DSBs truly reflects the map of COs along the genome, and what chromosomal features may influence the choice of DSB repair pathway.

Several factors affect CO formation and their sites of action may reflect how a DSB is repaired. A group of proteins collectively termed “ZMM” is necessary for the formation of about 85% of all COs in budding yeast [12], [13]. During yeast meiosis, the ZMM proteins act by stabilizing the Single End Invasion (SEI) recombination intermediate, which once formed is transformed via capture of the second break end into a double Holliday junction (dHJ) that is mainly resolved as a CO [12], [14], [15]. The ZMM group comprises proteins that act directly on recombination intermediates *in vitro*, such as the Mer3 helicase, which promotes D loop extension and the Msh4–5 heterodimer, which stabilizes dHJs. This group also includes Zip1, the central element of the synaptonemal complex (SC), as well as Zip2, Zip3, Zip4 and Spo16 that might promote SC formation through Zip1 polymerization between homolog axes [13], [16]. Currently, it is hypothesized that the ZMM proteins, by promoting SC initiation and by directly acting on recombination intermediates, protect the CO-prone recombination intermediates (dHJ) from dissolution by anti-CO proteins, such as Sgs1 [17]. Zip3 has orthologs in *C. elegans* (ZHP-3) and in mammals (RNF212) and is considered to be a SUMO E3 ligase that sumoylates chromosome axis proteins, thus promoting SC polymerization. Indeed, the Zip3 sequence includes a SUMO Interacting Motif (SIM) and a C3H2C3 Ring-Finger Motif (RFM) that are important for Zip3 *in vitro* E3 ligase activity and necessary for SC polymerization and correct sporulation [18].

Indirect evidence suggests that ZMMs localize at CO-designated sites, but this has never been demonstrated. ZMMs form foci during meiotic prophase at the time of recombination [16], [19], [20] and the number of Zip3 foci is compatible with CO frequency in wild-type yeast strains [20]. Moreover, in hypomorphic *spo11* mutant strains in which the number of DSBs but not of COs is reduced (a phenomenon known as CO homeostasis), the number of Zip3 foci follows the CO variation [21]. Finally, Zip2 foci are non-randomly distributed along chromosomes, like COs [22]. Among the ZMMs, Zip3 seems to be acting earlier because it is required for focus formation of all the other ZMMs [16].

We thus mapped Zip3 binding sites along individual genomic regions and genome-wide during budding yeast meiosis and then determined the features that influence its distribution. We show that Zip3 association with chromosomes is dynamic, occurring first with centromeres, in a DSB-independent manner, then with meiotic chromosome axes upon DSB formation and finally with DSB sites upon joint molecule formation, the preferred intermediate for CO production. These features establish Zip3 as a marker of CO-designated sites. Genome-wide mapping of Zip3 recruitment to DSB sites demonstrates the existence of different types of DSB hotspots based on CO production.

## Results

### Zip3 associates with centromeres early in meiosis, then with chromosome axes and finally with double-strand break sites

**Figure 1:**
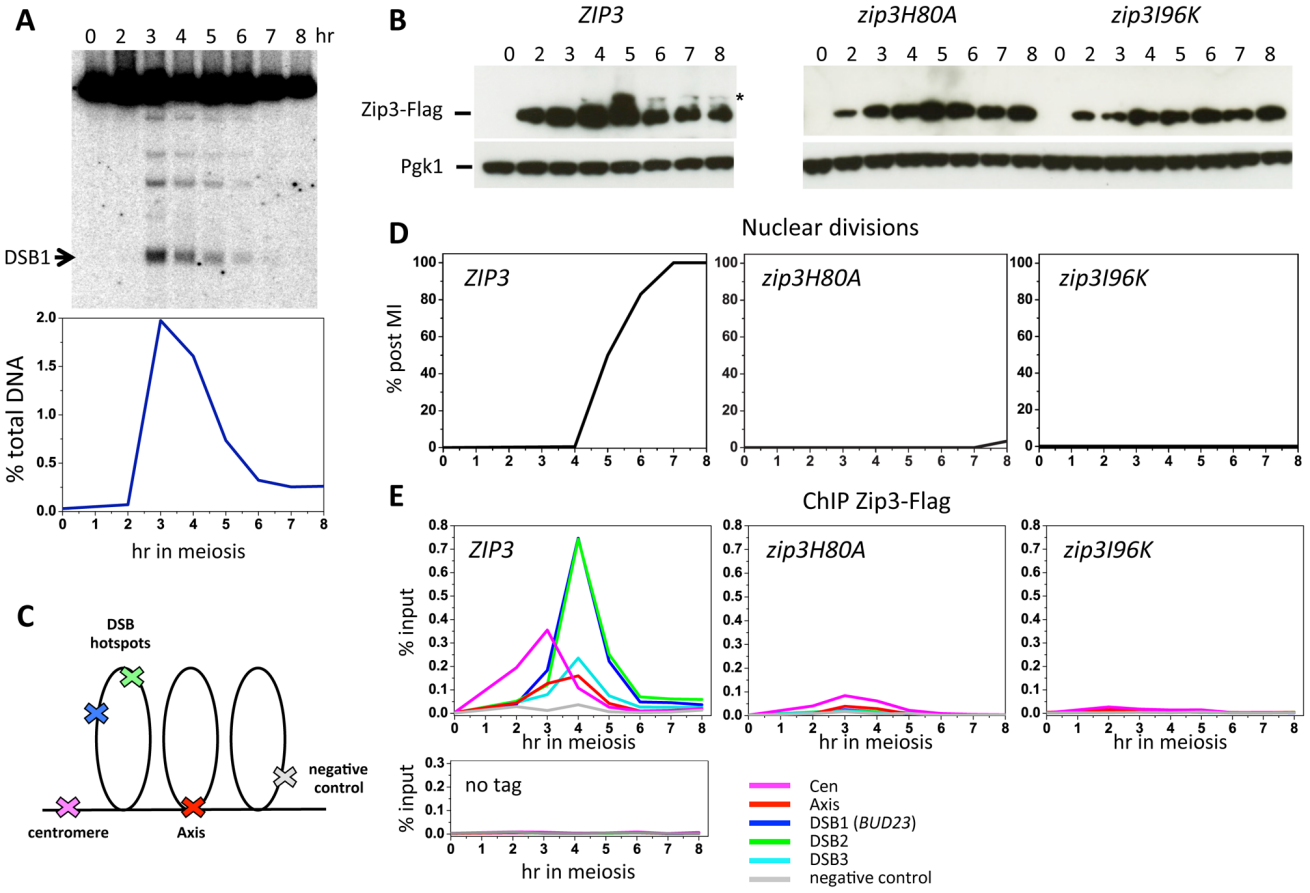
Zip3 SUMO ligase activity is required for Zip3 association with centromeres, axes, and meiotic double-strand break sites. (A) DSB formation in a wild-type (ORD9670) meiotic time-course at the DSB site in the *BUD23* promoter (DSB1), also monitored by ChIP in (E). The graph shows the quantification of DSB formation at DSB1. (B) Zip3-Flag expression was monitored by western blotting with an anti-Flag antibody in strains containing Flag-tagged wild-type *ZIP3* (ORD9670), *zip3H80A* (VBD1072) or *zip3I96K* (VBD1073) alleles. The asterisk indicates the presumed sumoylated forms of Zip3 [18]. Pgk1 served as loading control. (C) Schematic representation of the position of the different regions assessed by qPCR for Zip3 binding. (D) Meiotic progression in the three Zip3 strains, monitored by nuclear division. (E) ChIP monitoring of Zip3-Flag association with the indicated regions during the same time-courses in cells with wild-type (ORD9670), *H80A* (VBD1072) or *I96K* (VDB1073) *ZIP3* alleles. Below is the control experiment performed by ChIP with the anti-Flag antibody in an untagged strain (ORD7339).

Zip3 localization was previously investigated only by indirect immunofluorescence on chromosome spreads. To investigate Zip3 localization on meiotic chromosomes at about 1-kb resolution, we used chromatin immunoprecipitation (ChIP) and qPCR and yeast strains in which Zip3 was C-terminally tagged at its endogenous locus with three copies of the Flag epitope. Strains expressing the ZIP3-His6-FLAG3 allele showed normal meiotic progression and spore viability (98%, 205 tetrads dissected), showing that the tagged protein is functional. During a meiotic time-course, DSBs monitored at the *BUD23* promoter hotspot on chromosome 3 form and reach a maximum at 3–4 hr, before getting repaired (Figure 1A). Zip3 showed a reproducible dynamic localization. It bound first to centromeres from 2 hr after meiosis induction and before DSB formation, then to axis-associated sites and finally to DSB sites, particularly at 4 hr (Figure 1E). At this time, DSB fragments, as detected by Southern blotting, started disappearing (Figure 1A), indicating that DSB ends were already engaged in homologous recombination repair.

As Zip3 might be a SUMO E3 ligase, we investigated whether interaction with SUMO regulated Zip3 binding to the different chromosomal structures. To this aim, we mutated the Zip3 SIM (*zip3I96K* mutant) or the RFM (*zip3H80A* mutant) motif. Both mutated proteins were timely induced during meiosis, but they lacked the characteristic lower migrating bands that correspond to sumoylated Zip3 [18] (Figure 1B). In both mutants, early Zip3 binding to centromeres was abolished (Figure 1E), consistent with the previous suggestion that Zip3 recognizes sumoylated proteins at centromeres [18]. Moreover, recruitment to axis-associated and DSB sites was also mostly abolished (Figure 1E) and meiotic progression was impaired in both *zip3* mutants (Figure 1D), similarly to what was observed in *zip3* null mutants (data not shown). These findings indicate that Zip3 SUMO binding and E3 ligase activities are essential for Zip3 association with chromosomes and all its functions in meiosis. SUMO binding could be directly involved in Zip3 recruitment to all these chromosome locations or indirectly, if required only for the initial Zip3 enrichment at centromeres, and if this is an essential step for the subsequent recruitment of Zip3 to axes and DSB sites.

**Figure 2:**
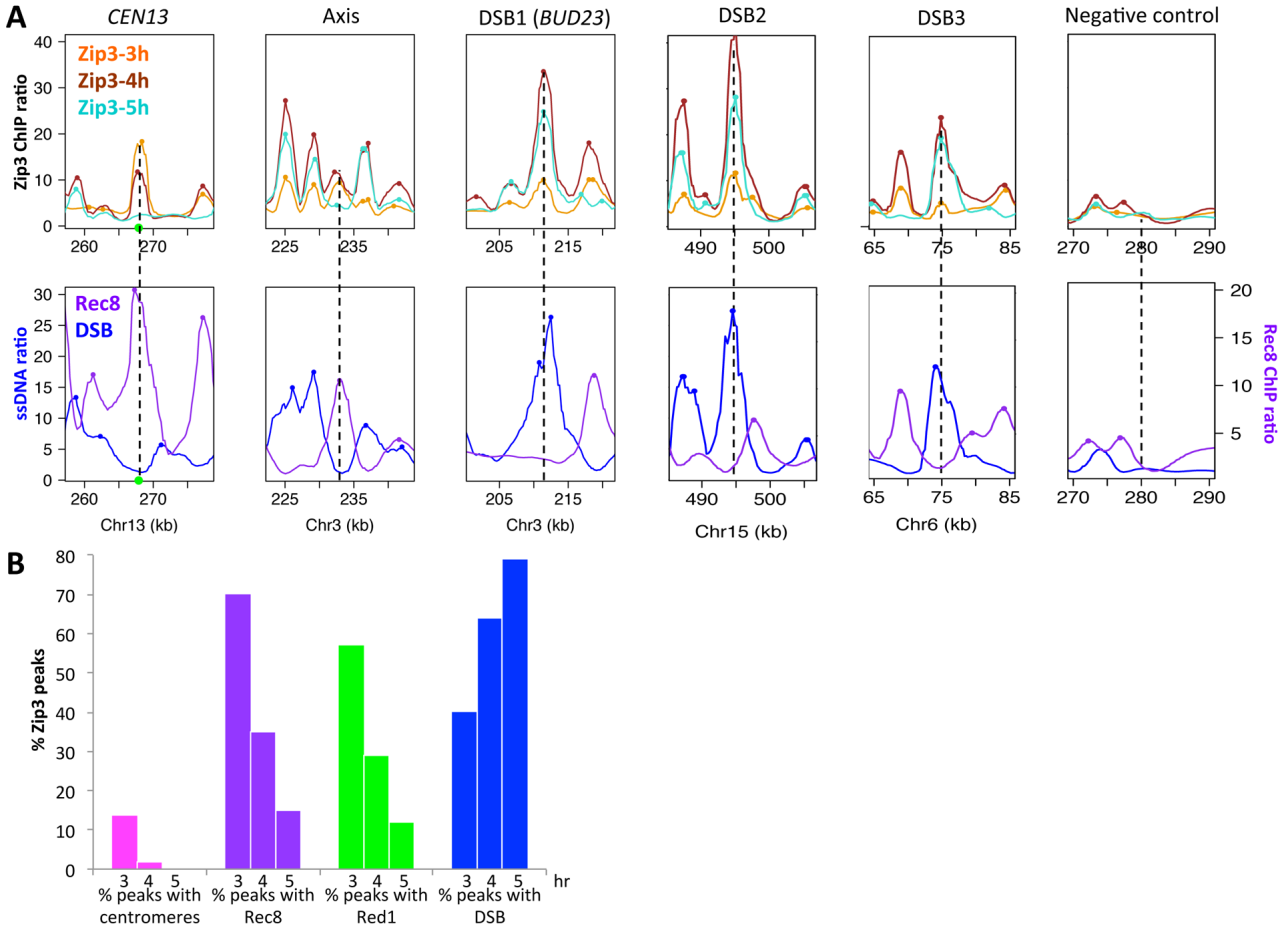
Genome-wide, Zip3 associates sequentially with different chromosomal structures. (A) Examples of Zip3 association with chromosomal regions during the meiotic time-course. The actual site is at the center of each×axis. Decile-normalized ratios are represented, after denoising and smoothing with a 2 kb window. Dots indicate sites were a peak was detected. The green circle indicates the centromere. Zip3-Flag data are from two independent time-courses of ORD9670 strain (see Figure S1). Rec8 data at 4 hr are from [23] and DSB data come from ssDNA signal that accumulate in *dmc1Δ* strains, from [3]. (B) Temporal variation of the specificity of Zip3 association with different chromosome features. The percentage of Zip3 peaks overlapping with each feature at the indicated time of meiosis is displayed. Values are detailed in Table 1, except for peaks with centromeres (peaks at less than 7.5 kb from a centromere).

We then mapped Zip3 binding sites genome-wide using microarrays at 3, 4 and 5 hr during meiotic progression in two independent meiotic time-course experiments (Figure S1A and S2). Genome-wide profiling confirmed the results obtained by ChIP and qPCR (Figure 2A). We then compared the Zip3 maps with the maps of the axis-associated Rec8 cohesin [23] and of Red1, another meiotic axis component that does not show the strong centromere association characteristic of Rec8 [24]. The reference DSB map was the map established by genome-wide mapping of ssDNA in a repair-defective *dmc1Δ* mutant [3].

At 3 hr after meiotic induction, Zip3 was strongly associated with centromeres, as seen on individual chromosomes (Figure 2A and Figure S3) and in the genome-wide analysis (Figure 2B, Figure S1B and Table 1). All 16 centromeres contained a strong Zip3 peak at less than 1 kb away, and 16% of the 287 Zip3 peaks at this time were found at less than 10 kb from the centromeres. Moreover, 81% of Zip3 peaks at less than 10 kb from a centromere overlapped with an axis-associated Rec8 peak and 38% with a Red1 binding site. At 3 hr, Zip3 was weakly associated with chromosome arms and the Zip3 peaks at more than 10 kb from a centromere coincided with Rec8 (54% peaks) and Red1 (50%) enriched sites (Figure 2B and Figure S3). This is reflected by the overall strong correlation between the Zip3 signal at 3 h and the Rec8 and Red1 profiles (Table 1). At 4 hr, Zip3 association with Rec8 sites diminished (only 35% of its 966 binding sites occurred at Rec8 sites), while its association with DSB sites started to increase (Figure 2B, Figure S4, and Table 1). Concomitantly, the relative Zip3 binding to centromeres decreased (Figure 2B). Finally at 5 hr, Zip3 was almost exclusively associated with DSB sites. Indeed, none of the 557 Zip3 peaks was found at less than 1 kb from centromeres and only 15% of Zip3 peaks coincided with a Rec8 peak at this time (Figure 2B and Table 1).

**Table 1:** Comparison of the ChIP–chip enriched peaks between pairs of experiments.

array1	array2	common peaks/peaks array1(% peaks array1)	Pcorr
Zip3 *spo11Δ*	Rec8	94/134 (70%)	0.74
Zip3-3h	Rec8	200/287 (70%)	0.67
Zip3-4h	Rec8	340/966 (35%)	0.19
Zip3-5h	Rec8	86/557 (15%)	0.05
Zip3 *spo11Δ*	Red1	60/134 (45%)	0.24
Zip3-3h	Red1	163/287 (57%)	0.47
Zip3-4h	Red1	284/966 (29%)	0.21
Zip3-5h	Red1	65/557 (12%)	0.06
Zip3 *spo11Δ*	DSB	26/134 (19%)	−0.11
Zip3-3h	DSB	114/287 (40%)	0.21
Zip3-4h	DSB	614/966 (64%)	0.64
Zip3-5h	DSB	438/557 (79%)	0.65
Rec8	Red1	547/728 (75%)	0.59
Rec8	DSB	88/728 (12%)	−0.17

The name of each experiment is indicated, as well as the number of peaks in common between the two experiments and the percentage of the peaks of the first experiment. Pcorr assesses the linear Pearson's correlation coefficient between the profiles of the two experiments after denoising and smoothing with a 2 kb window.

Thus, during meiosis, Zip3 associates first with centromeres. Centromeric Zip3 enrichment is then progressively reduced, whereas association with axis sites and particularly with DSB sites increases, in agreement with its previously described role in recombination.

### Centromeric Zip3 enrichment is independent of DSB formation

**Figure 3:**
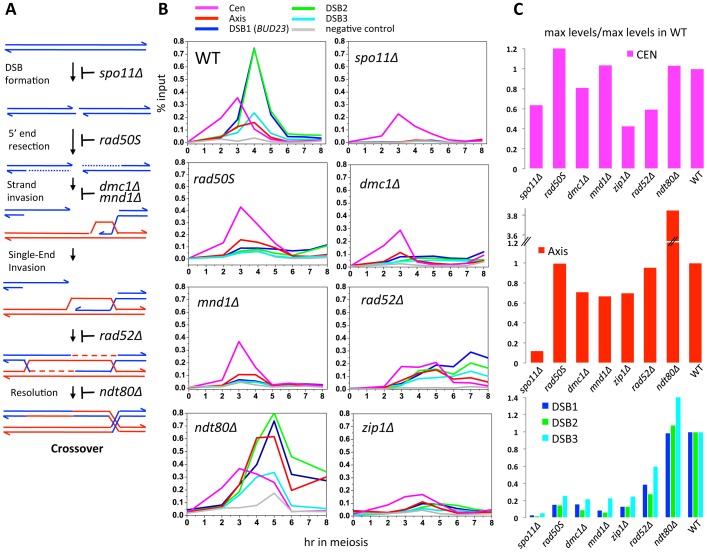
Formation of dHJs is required for full Zip3 recruitment to recombination sites. (A) Schematic of meiotic DSB repair and steps affected in the different mutants tested. For simplicity, only the pathway leading to dJH formation and CO resolution is represented. (B) Mutant analysis of the genetic requirements for Zip3 association with different chromosomal regions. In all experiments, cells from a synchronous time-course were processed for ChIP of Zip3-Flag and the association of Zip3 quantified by qPCR using primers that cover the indicated regions. All experiments were performed at least twice and gave similar results. WT: ORD9670; *spo11Δ*: ORD9684; *rad50S*: ORD9688; *dmc1Δ*: ORD9699; *mnd1Δ*: VBD1087; *rad52Δ*: VBD1108; *ndt80Δ*: VBD1001; *zip1Δ*: ORD9689. (C) Maximum Zip3-Flag enrichment levels observed in the mutants relative to the maximum levels observed during a wild-type time-course. Data are from those presented in (B).

To investigate which events triggered these dynamic changes in Zip3 localization we used yeast mutants that affect precise steps of recombination (Figure 3A). Zip3 association with centromeres early in meiosis might occur independently of DSB formation. Indeed, by using the *spo11Δ* mutant in which DSBs are not formed, we could show that Zip3 associated transiently with centromeres, but not with axis or DSB sites (Figure 3B and 3C: ChIP and qPCR analysis of individual sites; Figure S3 and Table 1: genome-wide analysis). Thus, association of Zip3 with centromeres is independent of DSB formation, whereas DSB formation is required for Zip3 association with the chromosome arms.

### DSB formation triggers Zip3 axis localization along chromosome arms

Moreover, in the *rad50S* mutant strain, where Spo11 DSBs are formed but not processed, Zip3 was recruited to centromeres and then chromosome axes, but not to DSB sites (Figure 3B and 3C). In the *dmc1Δ* mutant that is resection-proficient but deficient in strand invasion, Zip3 was transiently recruited to the axis-associated sites, with kinetics similar to those of wild-type cells, but associated rarely with DSB sites (at least eight times less than in wild-type cells), at the three sites examined (Figure 3B and 3C). Similarly, in the *mnd1Δ* mutant in which Dmc1 is loaded onto DSB ends but strand invasion does not occur [25], Zip3 was recruited to axes, but not to DSB sites (Figure 3B and 3C). We conclude that DSB formation is sufficient to trigger Zip3 localization at axis sites, whereas strand invasion is required for Zip3 association with DSB sites.

### Formation of dHJs is required for full Zip3 recruitment to recombination sites

In meiosis, *rad52Δ* mutants allow strand invasion by Dmc1 filaments, and wild-type levels of the Single End Invasion (SEI) intermediate, a crossover-specific intermediate, but are strongly impaired in the following step, second end capture, which leads to double Holliday junction formation and crossover resolution [26], [27]. In *rad52Δ* mutants, we detected centromere and axis association delayed but to nearly wild-type levels, but a strongly reduced binding of Zip3 to the three DSB sites (Figure 3B and 3C). This suggests that Zip3 requires the second end capture step, a crossover specific event, for associating with sites of DSB.

Finally, we analyzed Zip3 association with chromosome structures in the *ndt80Δ* mutant in which dHJs are formed but not resolved [14]. Zip3 recruitment to DSB sites occurred, at levels even higher than in wild-type, suggesting that dHJ formation is the event that triggers or stabilizes Zip3 recruitment to DSB sites (Figure 3B and 3C). In addition, we reproducibly detected a very strong enrichment on the axis, perhaps a consequence of the aberrant turnover of dHJ intermediates in this mutant. Finally, we noticed that Zip3 remained bound with DSB sites longer than in wild-type (Figure 3B).

This mutant analysis reveals that Zip3 associates with DSB sites only when they are engaged in dHJ intermediates, which are the CO precursors. Therefore Zip3 association with DSB sites can be considered as a marker for CO sites.

### Zip3 localization at DSBs requires Zip1

We next investigated the role of Zip1, which is the central element of the SC and was previously described as not necessary for Zip3 focus formation [16], [20], in Zip3 localization by ChIP and qPCR analysis. In the absence of Zip1, Zip3 was recruited to centromeres, although less than in wild-type cells, and to axis-associated sites, but only rarely to DSB sites (about 10-fold reduction, Figure 3B and 3C). This may be linked to the suggested role of Zip1 in stabilizing the Smt3 chains that are good binding substrates for Zip3 ([18] and Discussion).

### Tel1/Mec1 consensus phosphorylation sites are important for efficient Zip3 recruitment to recombination intermediates and for full levels of crossovers

**Figure 4:**
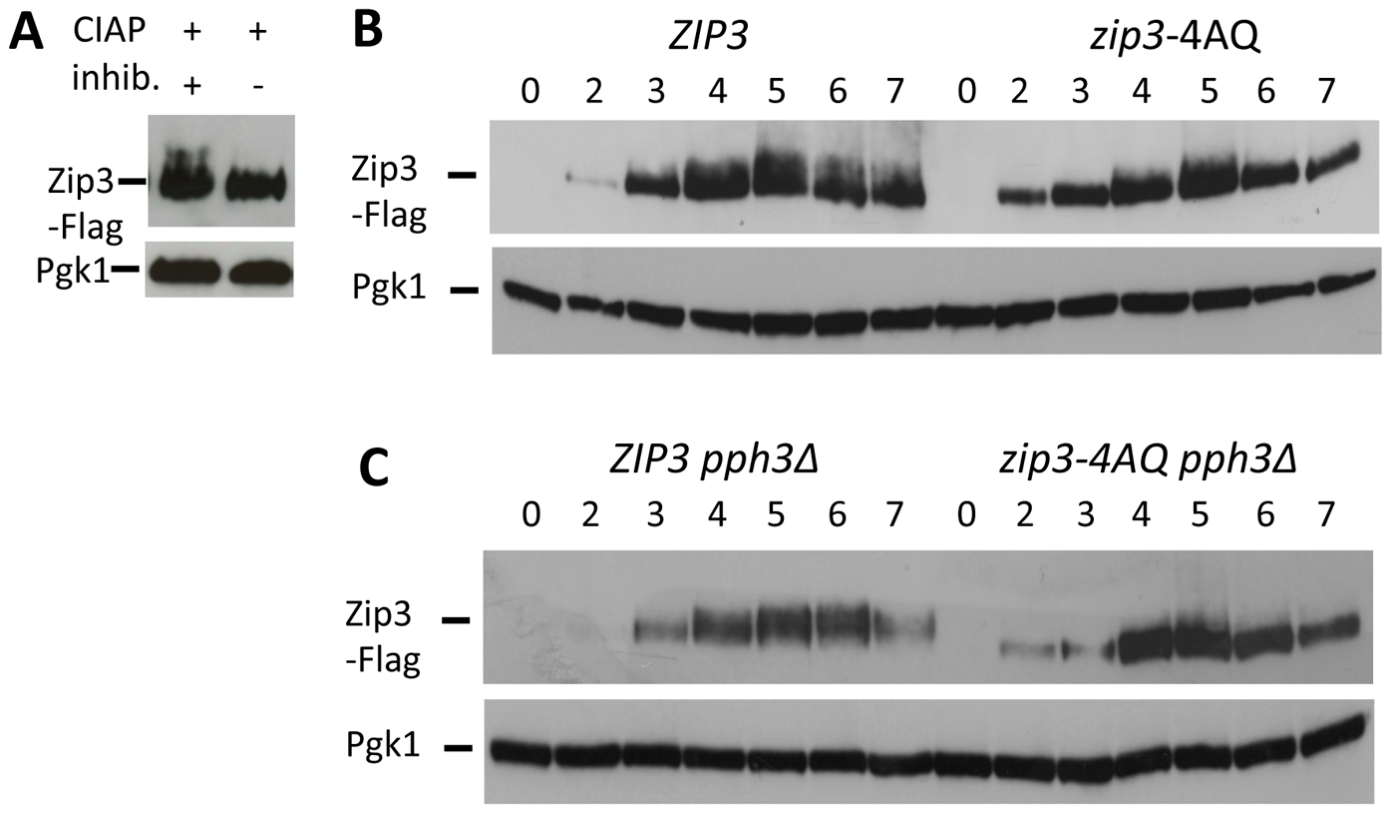
Zip3 phosphorylation depends on one or more S/T-Q Tel1/Mec1 kinases consensus phosphorylation sites and persists in a *pph3Δ* phosphatase mutant. (A) Migration shift of Zip3-Flag upon treatment with phosphatase. Total cell lysates from wild-type cells (ORD9670) at 5 hr in meiosis were treated with Calf Intestine Alkaline Phosphatase (CIAP) in the presence or absence of inhibitor. Proteins were separated and Zip3-Flag revealed by western blotting with an anti-Flag antibody. Pgk1 served as loading control. (B) Zip3-Flag expression in wild-type (ORD9670) and *zip3-4AQ* mutant (VBD1094) cells during a meiotic time-course analyzed by western blotting as in (A). (C) Zip3-Flag expression in *ZIP3 pph3Δ* (VBD1255) and *zip3-4AQ pph3Δ* (VBD1254) mutants during a meiotic time-course analyzed by western blotting as in (A).

Key events of meiosis are regulated by several kinases that are activated at different steps of meiosis. As Zip3 is phosphorylated in a DSB-dependent manner in meiosis ([18] and Figure 4A), we asked whether the dynamic Zip3 localization on chromosomes could be regulated by changes in its phosphorylation status. The CDK kinase Cdc28, together with the Cdc28-associated cyclins Clb5 and Clb6, is necessary for meiotic replication, DSB formation and SC formation [28] and can phosphorylate Zip3 *in vitro* [29]. *In vivo*, post-translational modifications of Zip3 are reduced in a *clb5* and *clb6* mutant [18], suggesting that Zip3 may be a CDK target. We mutated the six S/T-P CDK consensus motifs of Zip3 to A-P motifs (Figure S5) and found that mutant and wild-type Zip3 were similarly recruited and that meiotic divisions and spore viability were unaffected (Figure S5 and data not shown), demonstrating that Zip3 phosphorylation by CDK has no role in normal meiosis.

We next investigated the role of Zip3 phosphorylation by the Tel1/Mec1 kinases, the budding yeast homologs of ATM/ATR. Tel1 and Mec1 are activated upon meiotic DSB formation and play important roles in several key meiotic processes, such as DSB end resection, inter-homolog recombination and regulation of meiotic prophase checkpoint [30]. To this aim, we mutated the four S/T-Q consensus motifs for Tel1/Mec1 to A-Q motifs (*zip3-4AQ* mutant). This led to a decrease of the low migrating forms of Zip3 due to phosphorylation (Figure 4B). Many of the Mec1-dependent phosphorylated proteins are substrates for the PP4 phosphatase, including histone H2A129 or the Zip1 protein in meiosis [31]. We found that the Zip3 lower migrating forms accumulated in a *pph3Δ* catalytic subunit PP4 phosphatase mutant, but not in a double *zip3-4AQ pph3Δ* mutant (Figure 4C). Together, these findings provide strong indication that Zip3 is phosphorylated by the Mec1/Tel1 kinases during meiosis.

**Figure 5:**
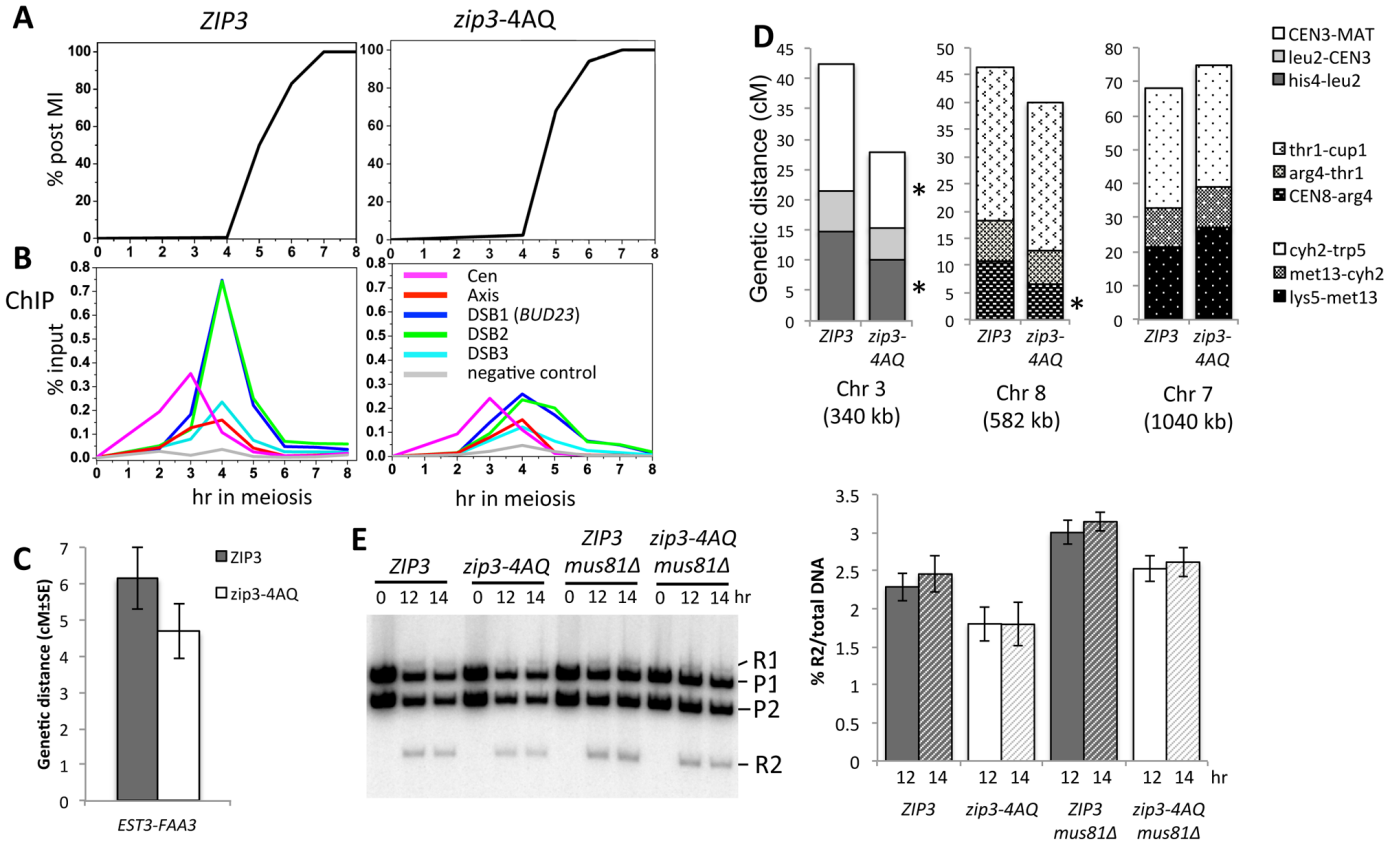
Mutation of the Zip3 consensus phosphorylation sites by Mec1/Tel1 kinases alters its association with DSB sites and decreases crossover levels. (A) Meiotic progression of wild-type (ORD9670) or *zip3-4AQ* mutant (VBD1094) cells. Nuclear divisions were monitored by DAPI staining. (B) Zip3 association in the time-courses shown in (A) was monitored by ChIP with anti-Flag antibodies and revealed by qPCR with primer pairs covering the indicated regions. (C) Genetic distance in the *EST3-FAA3* interval on chromosome IX measured in the *ZIP3-*Flag (VBD1229) and *zip3-4AQ-*Flag (VDB1113) strains (see also Table S2). The configuration of the hemizygous resistance markers used to measure the genetic distances is shown in Figure S8. (D) Genetic distances determined for nine intervals distributed on three chromosomes. See also Table S1. *ZIP3*-Flag: VBH334/VBH335 strain; *zip3-4AQ*-Flag: VBH332/VBH331 strain. (E) Physical analysis of COs in the *EST3-FAA3* interval in *ZIP3*, *zip3-4AQ* and *mus81Δ* mutants. Genomic DNA was extracted at the indicated times of synchronous meiosis and digested with *Bsp*EI and *Bss*HII. The parental (P1 and P2) and CO (R1 and R2) bands are indicated. The R2 band was quantitated and expressed as % of total DNA. *ZIP3*: VBD1229; *zip3-4AQ*: VBD1113; *ZIP3 mus81Δ*: VBD1244; *zip3-4AQ mus81Δ*: VBD1245. The graph indicates the mean of two independent experiments. Error bars represent standard deviation.

We next investigated the meiotic phenotypes of the *zip3-4AQ* mutant. Meiotic progression, spore viability (97%, 149 tetrads) and kinetics of DSB formation and repair were as in wild-type (Figure 5A and data not shown). At centromeres and axis sites, Zip3-4AQ was normally recruited. However, at the three tested DSB sites, loading of mutant Zip3 was 2- to 3-fold reduced in comparison to wild-type Zip3 (Figure 5B). Thus, the Mec1 consensus phosphorylation sites of Zip3 are important for its localization or stabilization on recombination intermediates. The reduced recruitment of Zip3-4AQ may result in lower CO frequencies. Indeed, in the *EST3*-*FAA3* interval flanking a strong DSB site on chromosome 9, fewer COs were formed in the *zip3-4AQ* mutant than in the wild-type *ZIP3* strain (Figure 5C and 5E). To test whether COs were reduced also at other loci, we performed tetrad analysis in a strain that contains genetic markers on chromosome 3, 7 and 8 to measure the genetic distances in three intervals per chromosome. Genetic distances were significantly reduced in three of the nine intervals tested, demonstrating the effect of the *zip3-4AQ* mutation on CO frequency (Figure 5D and Table S1). The observation that the genetic distance was reduced at two intervals on chromosome 3 (the smallest chromosome tested) and at none on chromosome 7 (the largest chromosome) suggests that perhaps smaller chromosomes are more affected by the Zip3 mutation (Figure 5D).

The residual association of Zip3-4AQ with DSB sites and the reduced CO frequency were still sufficient to promote full spore viability. We thus investigated whether the Zip3 S/T-Q motifs become essential for spore viability when DSBs are reduced. However, a mutant with reduced DSB levels did not show increased spore lethality when combined with the *zip3-4AQ* mutant (Figure S6). Finally, we hypothesized that the features of part of the COs in the *zip3-4AQ* mutant and of COs associated with wild-type Zip3 may be different. We thus measured CO frequency in the *mus81Δ* strain (wild-type Zip3), in which the alternative CO pathway is inactivated [32], and in the double *zip3-4AQ mus81Δ* mutants by physical analysis of the *EST3-FAA3* DSB site with flanking markers. In our hands and at the hotspot examined, mutation of *MUS81* did not affect CO formation in both strains, and CO was even slightly stronger in each case compared to its *MUS81* counterpart (Figure 5E). We conclude that mutating Mec1/Tel1 consensus phosphorylation sites of Zip3 decreases its association with DSB sites and reduces CO frequency, and that the remaining CO are not dependent on the *MUS81* pathway.

### Differential loading of Zip3 to DSB sites is indicative of the propensity of a DSB to be resolved as a crossover

In wild-type meiosis, Zip3 loading was not comparable at all DSB sites (see Figure S4). Specifically, although there was a high correlation between DSB and Zip3 sites at 4 and 5 hr after meiotic induction, Zip3 was enriched at DSB sites to various degrees (Figure S7).

**Figure 6:**
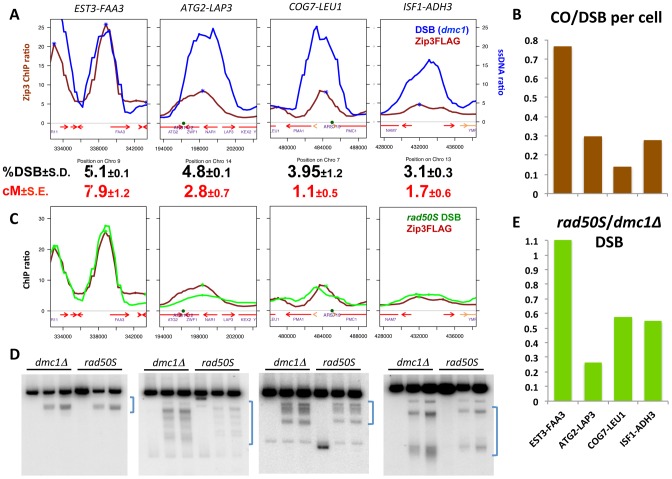
Variation in Zip3 binding to DSB sites reveals variation in crossover frequencies. (A) Analysis of DSB frequencies and genetic distances in intervals chosen from the genome-wide Zip3 map based on their strong (*EST3*-*FAA3*) or low (*ATG2*-*LAP3*, *COG7*-*LEU1*, *ISF1*-*ADH3*) Zip3 enrichment. Graphs represent decile-normalized data for Zip3 ChIP-chip at 4 hr or ssDNA at DSBs after denoising and smoothing with a 2 kb window. DSB frequencies in *dmc1Δ* strains are from flanking marker-containing strains and are the mean of two independent experiments (*EST3-FAA3*: VBD1168; *ATG2-LAP3*: VBD1218; *COG7-LEU1*: VBD1172; *ISF1-ADH3*: VBD1170). Genetic distances were determined by scoring the segregation of hemizygous resistance markers that flank each interval (see Table S2 and details of the intervals in Figure S8 and S9). (B) Comparison of the frequency at which a DSB is accompanied by a CO in each interval, assuming that only one chromatid per cell is cut in the interval, using the data presented in (A). (C) Graphs represent decile-normalized data for wild-type Zip3 ChIP-chip at 4 hr or *rad50S* covalently bound Spo11-HA ChIP-chip data (raw data from [3]), after denoising and smoothing with a 2 kb window, for the same intervals as in (A). (D) Comparison of DSB formation in the *dmc1Δ* and *rad50S* backgrounds at the high-Zip3 (*EST3-FAA3*) DSB site or the low-Zip3 (*ATG2-LAP3*, *COG7-LEU1* and *ISF1-ADH3*) DSB sites. Genomic DNA was extracted at 0, 5 and 6 hr during meiosis (from left to right) from *dmc1Δ* (ORD9699) or *rad50S* (ORD9688) cells and analyzed by Southern blotting. The brackets on the right of each panel indicate the physical interval comprised between the genetic recombination markers used to measure genetic distances. The extra band in the time 0 hr at the *COG7*-*LEU1* locus is likely due to star activity of the restriction enzyme used. (E) Ratio of DSB frequencies measured in a *rad50S* strain (ORD9688) over those measured in a *dmc1Δ* (ORD9699) strain in each interval.

To test whether variations in Zip3 loading at DSBs correlated with changes in recombination frequencies, we chose DSB sites with differential Zip3 binding and flanked them with hemizygous recombination markers (Figure S8) to assess both DSB and CO frequencies. In the wild-type strain, we chose a DSB site with strong Zip3 enrichment (*EST3-FAA3*) and three sites with relatively lower Zip3 accumulation (*ATG2-LAP3*, *COG7-LEU1* and *ISF1-ADH3*) (Figure 6A and Figure S7). The introduction of the flanking markers slightly lowered the DSB frequency in the interval (Figure S9) and we thus compared CO and DSB frequency in strains containing the flanking markers (Figure 6A and 6B and Table S2). The CO/DSB ratio varied among the sites and paralleled their relative Zip3 enrichment as measured on the ChIP-chip profiles: the three low-Zip3 DSB sites showed between 2.5 and 5 times less COs per DSB than the *EST3-FAA3* DSB site (Figure 6A and 6B).

To investigate whether such differential loading could be observed also in a situation where the DSB profile and number were changed, we compared the genome-wide maps of DSBs and Zip3-Flag binding sites in the *set1Δ* strain, in which DSBs are reduced and redistributed to new sites [33]. ChIP followed by qPCR indicated that Zip3 localized at DSB sites at 6 h and 7 h after meiotic induction, as expected because DSB formation is delayed by about 2 hours in this strain [33] (Figure S10A). Conversely and like in the wild-type strain, few Zip3 binding sites coincided with Rec8 sites at the 6 and 7 h time-points (Figure S10B and S11). Moreover, like in wild-type cells, Zip3 loading onto DSB sites was variable. For instance, *PES4*, a strong *set1Δ* DSB site, was highly enriched in Zip3, whereas *ARG3*, another strong *set1Δ* DSB site, was not (Figure S10C). We flanked each of these two sites by hemizygous markers (Figure S8) and measured crossover frequencies. Similarly, like in the wild-type strain, the high-Zip3 *PES4* site showed 2.2 times more COs per DSB than the low-Zip3 *ARG3* site (Figure S10C).

These results are consistent with a positive effect of Zip3 loading on DSB repair by CO and shows that in the genome, there are DSB sites that are less bound by Zip3 and less frequently repaired by CO than the average.

### High- and low-Zip3 DSB sites have distinct properties

**Figure 7:**
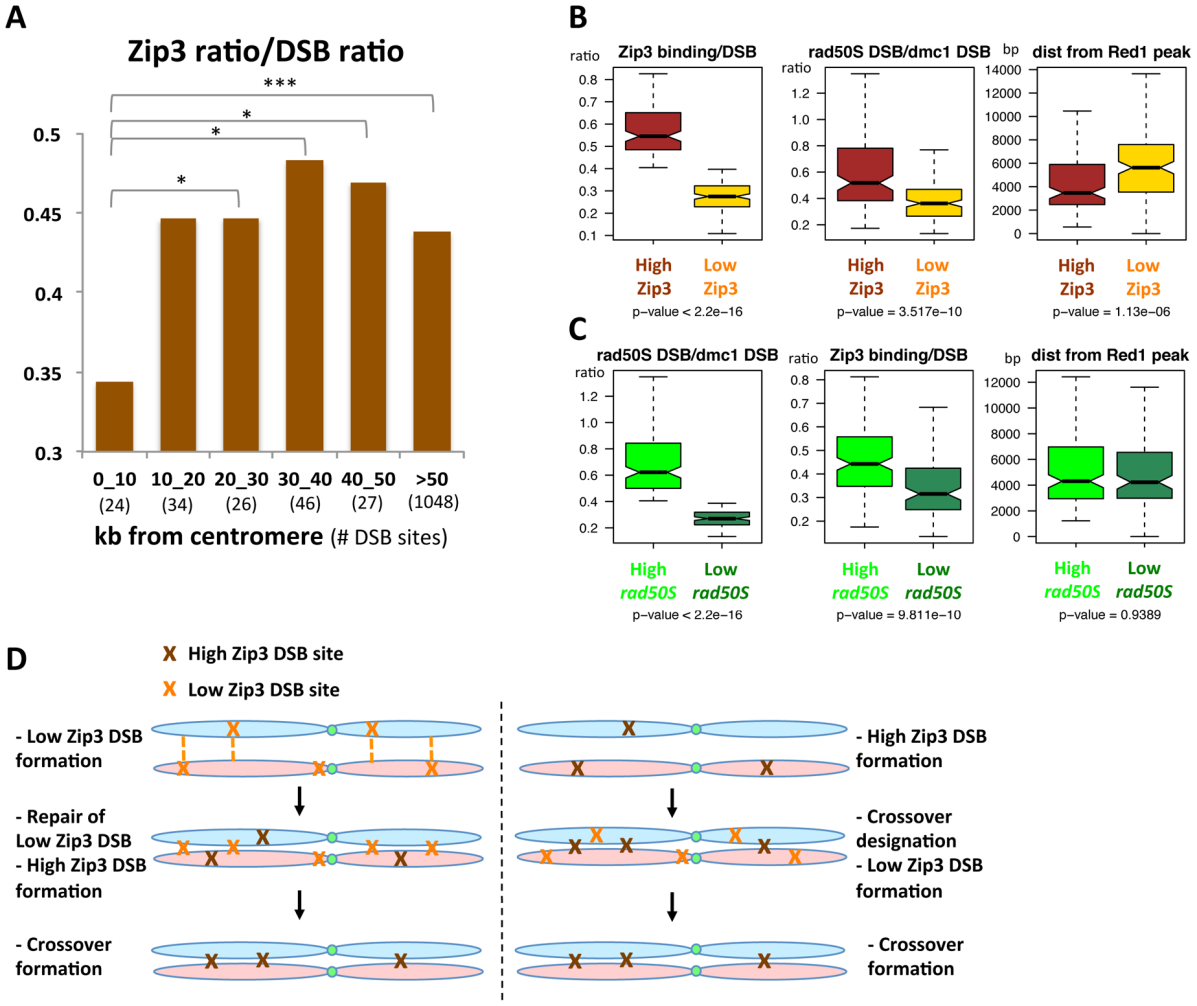
DSB sites with relatively high or low Zip3 enrichment differ in their distance from a centromere, in their DSB frequency in the *rad50S* mutant, or in their distance from an axis-association site. (A) Variation of the relative Zip3 binding to DSB sites relative to the distance from the centromere. At each DSB site in the considered distance interval from a centromere, the ratio of the Zip3 ChIP-chip signal at 4 hr was divided by the ssDNA ratio. Values are the mean of the values for all DSB sites in each interval (number between brackets). *: p<0.05 and ***: p<0.001 after Wilcoxon test. (B) Analysis of the indicated features at “High-Zip3” or “Low-Zip3” DSB sites (see details in the text). Boxplots indicate median (line), 25^th^–75^th^ percentile (box) ±1.5 times the interquartile range (whiskers). Non-overlapping notches of two boxes are indicative that the two medians are statistically different. p value indicates the result of a Wilcoxon test between the two DSB populations. The *rad50S* and *dmc1Δ* DSB datasets are from [3]. Red1 binding data are from [24]. (C) Analysis of the indicated features at “High *rad50S*” or “Low *rad50S*” DSB sites (see details in the text). Boxplots as in (B). p value indicates the result of a Wilcoxon test between the two DSB populations. The *rad50S* and *dmc1Δ* DSB datasets are from [3]. Red1 binding data are from [24]. (D) Model for the role of the Low Zip3 DSB. Two alternatives are proposed for the function of the Low Zip3 DSB. On the left panel, Low Zip3 DSBs are used for homolog pairing, before the High Zip3 DSBs are chosen for crossover. On the right panel, High Zip3 DSB are formed first and are the ones designated for crossover, the Low Zip3 occur later and may be used in case not enough early DSB engaged in crossover (see Discussion).

We then asked whether specific chromosome features were associated with these variations in Zip3 binding at DSB sites. We first investigated Zip3 loading at DSB sites close to centromeres as it was reported that inter-homolog CO frequency is usually low close to centromeres, although DSBs can form close to centromeres [7]. On several chromosomes, Zip3 did not bind to centromere-proximal DSBs (Figure S12) and, on average, the relative Zip3 signal at DSB sites close (less than 10 kb) to centromeres was significantly lower than in the rest of the genome (Figure 7A).

To extend the analysis beyond centromere regions, we defined from our mapping data two categories of DSB sites. Among the 400 strongest DSB sites previously determined in the resection-proficient *dmc1Δ* strain (without DSBs at less than 10 kb from a centromere), we identified “low-Zip3” DSB sites (n = 166 sites) and “high-Zip3” DSB sites (n = 142 sites) (see Protocol S1 for details on the classification). In these two DSB populations, the mean DSB signal was not statistically different (Wilcoxon test, p = 0.13). Similarly, several chromosome features, such as distance from a telomere or a centromere, and replication timing, were also not different (not shown). However, the strength of DSB signal measured in the resection-defective *rad50S* mutant was lower at low-Zip3 DSB sites than at high-Zip3 DSB sites [3] (Figure 6C and Figure 7B). Analysis of DSB formation by Southern blotting at the three low-Zip3 DSB sites *ATG2-LAP3*, *ISF1-ADH3* and *COG7-LEU1* (Figure 6D and 6E) and the low-Zip3 *set1Δ* DSB site *ARG3* (Figure S10D) confirmed that at these sites fewer DSBs were detected in the *rad50S* than in the *dmc1Δ* background. By contrast, the high-Zip3 *EST3-FAA3* and the high-Zip3 *set1Δ PES4* DSB sites showed similar DSB frequency in both backgrounds (Figure 6D and 6E and Figure S10D).

When we classified the DSBs in the *rad50S* mutant as high (157 sites) or low (113 sites), based on the peak signal intensity like we did for the Zip3 peaks, we found that the Zip3 signal was significantly lower in low-*rad50S* DSBs (Figure 7C). Overall, 66 DSB sites were present both in the low-Zip3 DSB and the low-*rad50S* DSB category, that is more than expected by chance (p<0.01, Pearson's Chi-square test). This further strengthens our observation that at least a subset of low-Zip3 DSB sites also shows reduced DSB formation in the *rad50S* mutant, suggesting that they have distinct properties.

The second chromosomal feature that varied between high- and low-Zip3 DSB sites was the distance from an axis-associated site, defined as a Red1 peak (Figure 7B). Low-Zip3 DSB sites were significantly more distant from an axis site than high-Zip3 DSB sites (median distance from a Red1 peak: 5599 bp and 3660 bp, respectively). Conversely, no difference in the distance from an axis-associated site was observed between low and high *rad50S* DSB sites (Figure 7C). Furthermore, the low-Zip3 DSB sites that were NOT low *rad50S* DSBs were still much further away from an axis site than the high-Zip3 DSB sites (5709 bp and 3660 bp from a Red1 peak, Figure S13).

We confirmed this observation in the *set1Δ* strain, in which the 200 strongest *set1Δ* DSB sites were classified as high- and low-Zip3 DSBs. High-Zip3 and low-Zip3 DSB sites did not show significant differences in their mean *dmc1Δ* DSB ChIP-chip signal (p = 0.66), but the low-Zip3 DSBs were significantly further away from a *set1Δ* Rec8 peak or a Red1 peak than the high-Zip3 DSB sites (Figure S10E).

Thus, we can distinguish two different categories of low-Zip3 DSB sites: sites with reduced DSB formation in the *rad50S* mutant and sites that are far from an axis-associated site, suggesting that proximity to an axis site favors DSB binding by Zip3 and resolution as a CO (see Discussion).

## Discussion

Here we show that the ZMM protein Zip3 interacts dynamically with chromosomes, associating first with centromeres, then with chromosome axes upon DSB formation, and finally with DSB sites on the recombination intermediates engaged in CO formation. We thus propose that Zip3 is a molecular marker of CO sites. We then demonstrate that Zip3 association with chromosomes requires its SUMO E3 ligase motifs, thus implying that SUMO recognition and transfer are needed for Zip3 interaction with chromosomal proteins. Zip3 phosphorylation sites by Mec1/Tel1 kinase are also important for Zip3 full loading on DSBs and CO formation. Finally, we show the existence of DSB sites that are rarely bound by Zip3 and that produce fewer COs than the average of DSB hotspots. These low-Zip3 DSB sites are sensitive to the effect of the *rad50S* mutation and tend to be away from an axis-association site, where the recombination process takes place.

A recent study showed that the proteins necessary for DSB formation reside on the chromosome axis, rather than at the sites of DSB formation in loop sequences [24]. This suggests that at the time of DSB formation, DSB hotspot sequences are already located on the chromosome axes. Indeed, using ChIP assays, we found that Zip3 first associates with axes and DSB sites, and later during the recombination process (when dHJs are formed at the pachytene stage) it becomes almost exclusively associated with DSB sites. We propose that at this stage, the recombination intermediates are located in the inter-homolog space and are detached from the axis, as previously seen cytologically in *Sordaria* [34]. Although recombination takes place close to the axis, axis-associated sites might be less immunoprecipitated by ChIP, because Zip3 is less intimately linked to these sites than to DSB sites.

Our ChIP analysis of Zip3 localization in yeast mutants that affect defined steps of recombination indicates that DSB formation is sufficient to trigger Zip3 localization at axis sites, whereas Zip3 associates with DSB sites only when they are engaged in dHJ intermediates. Our results are in apparent contrast with previous cytological findings about Zip3 foci in various mutants. In the *rad50S* mutant, many Zip3 foci co-localized with Mre11, which associates with DSB sites in this strain [20], [35]. However, we found that Zip3 does not associate with DSB sites in this mutant. The previously described foci could correspond to Zip3 loading on chromosome axes where Mre11-enriched DSB sites may also be located in the *rad50S* mutant. Similarly, Zip3 foci were previously detected in the *dmc1Δ* mutant [36], whereas in our study Zip3 was normally associated with axis sites, but very little with DSB sites. This was not due to experimental artifacts due to a differential ability to immunoprecipitate Zip3 in these mutants, since we observed constant Zip3 recovery during the whole time-courses after immunoprecipitation (data not shown). These discrepancies underscore the complementarity between ChIP approaches and cytological studies and show that similar patterns of foci can underlie completely different protein localizations along chromosomes, as revealed by our study.

The early Zip3 association with axes following DSB formation could be due to Zip3 binding to cleaved DSB sites that are located on the axis, or to a generalized recruitment of Zip3 on chromosome axes, maybe through interaction with a protein phosphorylated upon DSB formation. Our ChIP-chip data favor the second explanation because axis sites close to strong DSB sites were not more enriched in Zip3 and Zip3 binding to axes was rather homogenous along chromosomes (data not shown). The protein responsible for Zip3 loading onto axis sites could be an axis protein that is phosphorylated by the Tel1/Mec1 kinases, such as Hop1 [37].

We observed a reduced recruitment of Zip3 to all chromosomal regions in the *zip1Δ* mutant. It was proposed that at centromeres, Zip1 stabilizes Smt3 chains, made by other SUMO ligases acting in early meiosis, thus favoring Zip3 binding to centromeres. Our data confirm previous cytological observations [38] and suggest that Zip3 loading at centromeres may be a consequence of Zip1 localization at centromeres early in meiosis. Indeed, Zip1 association with centromeres is Zip3-independent and early centromere coupling mediated by Zip1 does not require Zip3 [39]. Our results in the *zip3* SUMO ligase and the *zip1Δ* mutants are consistent with a previously proposed model [18]: after the initial Zip3 recruitment to DSBs, which requires its SUMO binding motif (our results), Zip1 binds to and stabilizes the Smt3 chains deposited by Zip3. This in turn induces a second wave of Zip3 recruitment to DSB sites via its SUMO binding motif [18]. Indeed, in the *zip1Δ* mutant, Zip3 association with DSB sites was strongly decreased.

Interestingly, Zip3 foci persisted more on DSB sites in the *ndt80Δ* mutant than in the wild-type. The *ndt80Δ* mutant accumulates non-cleaved dHJs and thus our data are consistent with the proposed role of Zip3 and the ZMM in general to stabilize the crossover-designated intermediates from D-loop dismantling and later from dHJ dissolution by activities exerted by anti-crossover factors such as Sgs1 [40]. Strikingly, Zip3 association with the axis site reached very high levels in *ndt80Δ* cells. This may be due to a change of structure within the synaptonemal complex that persists in this mutant and that alters the association of sites undergoing dHJ with axis-associated sites, and renders these closer to strong DSB sites and thus more closely associated with Zip3. It would be interesting to determine if this increase of Zip3 association is seen for all axis-associated sites or only those that are close to strong DSB sites.

We detected a negative influence of the centromere on the relative binding of Zip3 to DSB sites. However, Zip3 binding was not abolished, although these regions show few CO and NCO events and have been suggested to repair their DSBs mostly using the sister chromatid [7]. A previous study showed that during DSB repair by sister chromatid recombination, the formation of associated joint molecules still depends on the ZMM protein Msh4 [2]. Similarly, we found that when a DSB is forced to be repaired using the sister chromatid, it still binds to Zip3, albeit to a lesser extent than when it is repaired by the homolog (unpublished results). Thus, DSBs might bind to Zip3 also very close to centromeres if they form joint molecules with the sister chromatid, explaining why we see residual Zip3 association with DSB in these regions.

In the rest of the genome, we detected qualitative differences among DSB sites. Specifically, for a chosen set of sites, we show that the CO frequency per DSB can vary from one DSB site to another and that this behavior can be predicted based on the relative Zip3 enrichment at the site. These DSB hotspots have peculiar properties: they form DSBs at a lower frequency in the *rad50S* mutant (our results and [3]) and they tend to overlap with coding regions (our results and [4]). Previous studies showed that in an artificially late replicating chromosomal region, meiotic DSBs also formed later. Interestingly, DSB formation at these sites is affected in *rad50S* mutants [41]. In the *rad50S* mutant DSB formation is impaired at many regions [3] and by extension these could be naturally late-occurring DSBs. Indeed, these “low-*rad50S*” DSBs tend to occur later, but the asynchrony of meiotic time-courses makes it difficult to reproducibly detect a delay ([3] and data not shown). Based on these data, we can hypothesize that the low-Zip3 DSBs that we have studied are naturally late-forming DSBs. This would imply that in a given chromosomal region, early-forming DSBs are the preferred substrate for CO designation. Indeed, CO designation is a very early event, much earlier than Zip3 association, which we defined as a CO marker in this study. Upon early DSB formation, the CO designation of one DSB might relieve the chromosomes from the experienced stress, thus locally disfavoring further CO designations and explaining CO interference [12], [42]. Thus, a DSB formed later in this region will have little chance to be chosen as a CO event. We also found that besides the *rad50S* effect on DSB frequency and the possibly associated differential timing of DSB formation, low-Zip3 DSBs are more distant from an axis-associated site. For their repair, and likely also for their formation, DSB sites interact with the chromosome axis, particularly where the Red1 and Hop1 proteins reside, and cytological studies showed that the association between Zip3 and Hop1/Red1 occurs prior to SC polymerization, likely at the future CO sites [36]. We propose that a DSB site away from the axis will be less efficiently brought or kept on the axis, making it less favorable for CO designation.

Our data have important implications for the control of meiotic recombination and genetic distances at the level of DSB formation and repair outcome. It will be interesting to investigate whether the DSB sites with low CO frequency we identified are NCO hotspots being repaired via the homolog or if they are repaired via the sister chromatid and whether they are preferential binding sites for anti-crossover activities. These extra-DSB sites rarely repaired as crossover may be either used early for homolog pairing, which precedes crossover formation, or conversely, they may be later “safety” DSB made in case insufficient early DSB go into crossover (Figure 7D).

Our work paves the way for further studies in other organisms, especially in mammals where the number of DSB largely exceeds that of COs.

## Materials and Methods

### Yeast strains and media

All yeast strains (Table S4) are derivative of the SK1 background. They were produced by direct transformation or crossing to obtain the desired genotype. Details of strain construction are in Protocol S1. All transformants were confirmed to have the flanking marker at the correct locus by PCR analysis to discriminate between correct and incorrect integrations.

Synchronous meiosis in liquid culture was performed as described [43]. Progression through meiosis was monitored by scoring nuclear divisions after DAPI staining.

### Western blot analysis

Western blotting was performed as described [23] using the mouse monoclonal anti-FLAG antibody M2 (Sigma, 1∶1000), except for detecting phosphorylated Zip3 (Figure 4 and Figure S6) where samples were separated in 10% 150∶1 acrylamide-to-bisacrylamide gels. Dephosphorylation assays were carried out as described [18], using calf intestinal alkaline phosphatase in the presence or not of 20 mM of the phosphatase inhibitor sodium orthovanadate.

### Tetrad analysis of recombination on chromosomes III, VII, and VIII

For genetic distances on chromosomes III, VII and VIII, haploids were mated at 30°C on YPD supplemented with 1% Adenine for 5 hr before replica-plating on solid sporulation medium (1% potassium acetate) and incubated at 30°C for at least 48 hr. For recombination between hemizygous drug resistance markers, diploids were grown on YPD plates and then replica plated on sporulation medium at 30°C for at least 48 hr. Asci were dissected on YPD supplemented with 1% Adenine and replica-printed to the appropriate media to check for marker status. P (parental), NPD (non-parental) and T (tetratype) were scored to calculate the genetic distances as described in [44]. For calculation of the map distance, standard error calculations were performed using the Stahl Lab Online tools (http://www.molbio.uoregon.edu/~fstahl/). For calculation of the ratio between CO and DSB per cell, we divided the % of cells that received a CO (genetic distance * 2) by the % of cells that received a DSB (%measured DSB frequency * number of chromatids per cell, i.e. 4).

### Physical detection of DSBs and COs by Southern blotting

Genomic DNA was prepared, analyzed and DSB or CO frequency was determined as described [23]. The used restriction enzymes and probes are in Protocol S1.

### Chromatin immunoprecipitation and real-time quantitative PCR

For each time-point, cells were processed and ChIP was performed as described [23], using 2 µg of the mouse monoclonal anti-FLAG antibody M2 (Sigma) and 30 µL Protein G magnetic beads (New England Biolabs). Quantitative PCR was performed using immunoprecipitated DNA or whole-cell extracts as described [23]. DSB1, DSB2 and DSB3 sites were chosen according to the genome-wide mapping of [3]. DSB1 is in the promoter of *BUD23* on chromosome 3; DSB2 is in the promoter of *ECM3* on chromosome 15 and DSB3 in the promoter of *RIM15* on chromosome 6. Axis site was chosen from the Rec8 binding data of [45], on chromosome 3. Negative control site is neither a DSB site nor a Rec8 site, and is located in the promoter of *CDC39*, on chromosome 3. Primer positions are in Protocol S1. All time-courses and ChIP assays were repeated at least twice from independent experiments and gave similar results.

### Microarray hybridization, data acquisition, and analysis

Immunoprecipitated DNA and whole-cell DNA were amplified, labeled and hybridized to Agilent 44 k yeast whole genome oligonucleotide arrays as described [33]. Microarray images were read using an Axon 4000B scanner and analyzed using the GenePix Pro 6.0 software (Axon Instruments). Files were converted to text files and analyzed using the R software. The signal intensities of profiles were normalized, by dividing all values by the mean of the lowest 10% ratio probes of the array (decile normalization, as described [24]). In this way, the 10% lowest values fall below 1, so that everything below and around this value can be interpreted as background. The resulting normalized data were next denoised and smoothed, as described before [23]. Raw data from [33], [3] and [24] were reanalyzed as described before [23]. Peaks were identified after denoising and smoothing with a 2 kb window (except for the data by [24], where a 300 bp window was used), and compared as described [23]. In the *set1Δ* Zip3-Flag 6 and 7 hr ChIP-chip assays, a very high signal was obtained, and we adjusted the threshold to 5 to obtain a number of Zip3 peaks comparable to that of the other experiments. High Zip3 DSB sites were DSB sites that coincide with a Zip3 peak the signal intensity of which differed by less than 50 ranks from that of the DSB site; Low Zip3 DSB sites were DSB sites either not bound by Zip3 or that coincide with a Zip3 peak the signal intensity of which was at least 100 ranks lower than that of the DSB site.

For the chromosome coordinates, we used the Saccharomyces Genome Database features (http://downloads.yeastgenome.org/curation/chromosomal_feature/) of the last update from July of 2010.

### Accession numbers

The ChIPchip data generated in this study have been deposited at the Gene Expression Omnibus database, accession number GSE40563. Processed data for all chromosomes are provided in Table S3.

## Supporting Information

Figure S1 Genome-wide ChIPchip analysis of Zip3 at 3, 4 and 5 h in meiosis. (A) qPCR analysis of the Zip3-Flag ChIP samples used for ChIP-chip analysis. Zip3 association was monitored at the indicated regions in a wild-type strain (ORD9670). The average values from two independent time-courses are shown. The three red arrows indicate the time-points that were used in our ChIPchip analysis. (B) Global temporal variation of Zip3 association with centromeres, axis-association sites and DSBs. For each category, the following regions were considered: centromeres (Zip3 signal at probes at less than 200 bp from a centromere), Rec8, Red1 and DSBs (Zip3 signal at the 200 strongest Rec8, Red1 and DSB peaks, respectively). The decile-normalized ratios after denoising and smoothing using a 2 kb window are indicated. Boxplots show the median (line), 25^th^–75^th^ percentile (box) ±1.5 times the interquartile range (whiskers). p value indicates the result of a Wilcoxon test between the two indicated time-points.
(TIF)

Figure S2 Genome-wide profiles of Zip3 localization. Average ChIP-chip Zip3-Flag decile-normalized ratios from two independent wild-type (ORD9670) meiotic time-courses are plotted after denoising and smoothing with a 1 kb window along the 16 chromosomes. Black circles indicate the centromere. Same experiment as in Figure S1.
(TIF)

Figure S3 Genome-wide profiles of Zip3 ChIP at 3 hr in meiosis, Zip3 in a *spo11Δ* mutant and Rec8 Flag. Average decile-normalized ratios are plotted along the 16 chromosomes after denoising and 1 kb window smoothing. Green circles indicate the centromere. Rec8 data are from [23]. Zip3-Flag at 3 hr like in Figure S2 and *spo11Δ* at 3 hr is from ORD9684 strain.
(TIF)

Figure S4 Genome-wide profiles of Zip3 ChIP at 4 hr and ssDNA accumulated at DSB ends in a *dmc1Δ* mutant (raw data from [3]). Average decile-normalized ratios are plotted along the 16 chromosomes after denoising and 1 kb window smoothing. Green circles indicate the centromere. Zip3-Flag at 4 hr like in Figure S2. Blue dots indicate DSB sites overlapping with a Zip3 peak.
(TIF)

Figure S5 Mutation of the Zip3 consensus phosphorylation sites for the CDK kinase has no effect on Zip3 association with DSB sites. (A) Zip3-Flag expression in a wild-type (ORD9670) and *zip3-6AP* mutant strain (VBD1093) during a meiotic time-course. Zip3-Flag was monitored by western blotting with an anti-Flag antibody. Pgk1 served as loading control. (B) Meiotic progression in the same time-courses as in (A). Nuclear divisions were monitored by DAPI staining. (C) Monitoring of Zip3 binding in the same time-courses as in (A) and (B) by ChIP with an anti-Flag antibody and revealed by qPCR using primer pairs that cover the indicated regions.
(TIF)

Figure S6 Spore viability in strains with reduced DSB formation and wild-type Zip3-Flag or mutant Zip34AQ-Flag. The proportion of 4, 3, 2, 1 or 0 viable spore per tetrad is indicated for each strain. *SPO11 ZIP3*: ORD9670 (205 tetrads); *spo11YF/HA ZIP3*: VBD1191 (124 tetrads); *spo11YF/HA zip3-4AQ*: VBD1192 (134 tetrads).
(TIF)

Figure S7 ChIP-chip profiles for Rec8, ssDNA and Zip3 around high- and low-Zip3 DSB sites. The actual site is at the center of each plot×axis. Decile-normalized ratios are represented, after denoising and smoothing with a 2 kb window. Dots indicate sites were a peak was detected. Same strains and experiments as in Figure 2.
(TIF)

Figure S8 Schematic representation of the hemizygous flanking marker configuration used to assess genetic distances.
(TIF)

Figure S9 DSB frequencies in the chosen high-Zip3 and low-Zip3 intervals in the absence or presence of hemizygous flanking markers. Genomic DNA was extracted at the indicated time during meiosis from *dmc1Δ* cells and analyzed by Southern blotting. The brackets on the side of each panel indicate the physical interval comprised between the genetic recombination markers used to measure genetic distances. Red arrows indicate new DSB due to the insertion of a flanking marker. Below each panel is indicated the DSB frequency measured from at least two independent time-courses ± standard deviation. *EST3-FAA3*: with flanking markers: strain VBD1168; no markers: VBD1172. *ATG2-LAP3*: with flanking markers: strain VBD1218; no markers: VBD1172. *COG7-LEU1*: with flanking markers: strain VBD1172; no flanking markers: VBD1168. *ISF1-ADH3*: with flanking markers: strain VBD1170; no flanking markers: VBD1172.
(TIF)

Figure S10 Zip3 associates with DSB hotspots in *set1Δ* with varying frequencies. (A) ChIP monitoring of Zip3-Flag association with the indicated regions during a meiotic time-course in *set1Δ* cells (VBD1005). (B) Comparison of the profiles between pairs of experiments. The name of each experiment is indicated, as well as the number of peaks in common between the two experiments and as percentage of the peaks of the first experiment. Pcorr assesses the linear Pearson's correlation coefficient between the profiles of the two experiments after denoising and smoothing with a 2 kb window. *set1Δ* DSB: raw data are from [33]; *set1Δ* Rec8: data are from [23]. (C) Comparison of Zip3-Flag binding and *dmc1Δ* DSBs in *set1Δ* strains in the *PES4* and *ARG3* regions, two sites with increased DSB frequency in the *set1Δ* mutant. *set1Δ* Zip3-Flag data are from the same time-course as in (A). *set1Δ* DSB raw data are from the Rpa ChIP-chip at 7 hr in a *set1Δ dmc1Δ* strain [33]. DSB frequencies were measured in *dmc1Δ* strains at 7 hr in meiosis (ORD9624) and values are from eight (*PES4*) and six (*ARG3*) independent experiments. Genetic distances were determined by scoring the segregation of hemizygous resistance markers flanking each interval (see Table S2 and details of the intervals in Figure S8). (D) Comparison of *set1Δ* DSB frequencies in the *dmc1Δ* (ORD9624) and *rad50S* (VBD1117) backgrounds at *PES4* and *ARG3* sites. DSB formation was measured by Southern blotting as described in Materials and Methods. Brackets indicate the interval in which genetic distances were measured. (E) Boxplot representation of the analysis of the indicated features at “High-Zip3” or “Low-Zip3” DSB sites (see details in the text). High-Zip3 DSB sites (n = 81) were selected among the strongest 200 *set1Δ* DSBs based on the presence of an associated Zip3 peak at 6 hr the signal intensity of which was less than 50 ranks away from that of the DSB site. Low-Zip3 DSB sites (n = 39) were selected among the strongest 200 *set1Δ* DSBs based on the absence of a Zip3 associated site, or on the presence of a Zip3 site with signal intensity at least 150 ranks below that of the DSB site. The *set1Δ* Zip3 at 6 hr, 7 hr, *dmc1Δ* DSB and Rec8 data sets are the same as in (B). Red1 binding raw data are from [24].
(TIF)

Figure S11 Genome-wide profiles of *set1Δ* ChIP-chip of Zip3 at 6 hr and RPA accumulated at DSB ends in a *set1Δ dmc1Δ* mutant (raw data from [33]). Decile-normalized ratios are plotted along the 16 chromosomes after denoising and 2 kb-window smoothing. Green circles indicate the centromere. Same experiment as in Figure S10C, with blue dots indicating DSB sites overlapping with a Zip3 peak.
(TIF)

Figure S12 Association of Zip3 with centromere-proximal DSBs. Examples of Zip3 and DSB signals at four centromere regions and one chromosome arm. Graphs represent decile-normalized data after denoising and smoothing with a 2 kb window of Zip3 ChIP-chip at 4 hr or ssDNA at DSB. Same data as in Figure 6A.
(TIF)

Figure S13 Features of the low-Zip3 DSB sites that are not low-*rad50S* DSBs (see details in the text). The *rad50S* and *dmc1Δ* DSB datasets are from [3]. Red1 binding data are from [24].
(TIF)

Protocol S1 Contains details about yeast strains construction, enzymes and probes used for DSB mapping and position of qPCR primers.
(DOC)

Table S1 Effects of the *zip3-4AQ* mutation on genetic distances in intervals along three chromosomes. Map distances and standard errors (in centiMorgans; cM) were calculated from parental ditypes (PD), non-parental ditypes (NPD) and tetratypes (T) as described in the Materials and Methods. *P* values are for *G* tests performed on parental ditype, non-parental ditype, and tetratype segregation patterns for pairwise comparison between wild-type *ZIP3-Flag* and the *zip3-4AQ-Flag* mutant. Same strains as in Figure 5D.
(XLSX)

Table S2 Genetic distances at High- and Low-Zip3 DSB sites.
(XLSX)

Table S3 Processed ChIP-chip data for Zip3-Flag in wild-type (ORD9670), *spo11Δ* (ORD9684) or *set1Δ* (VBD1005) strains and for untagged control (ORD7339). Average decile-normalized ratios calculated from two independent experiments for each condition are included, as well as denoised ratios after smoothing with a 2 kb window. The RefNumber column shows the reference provided by the manufacturer (Agilent) to allow easy alignment with characteristic features or other data using the same microarray platform. The raw and processed data have been deposited in the Genome Omnibus Database (GSE40563).
(TXT)

Table S4 Strains used in this study.
(DOC)
