# Supplementary figures and images for: Differential Association of the Conserved SUMO Ligase Zip3 with Meiotic Double-Strand Break Sites Reveals Regional Variations in the Outcome of Meiotic Recombination
Source: PLoS Genet. 2013 Apr 4;9(4):e1003416. doi: 10.1371/journal.pgen.1003416 (PMC3616913; doi:10.1371/journal.pgen.1003416)

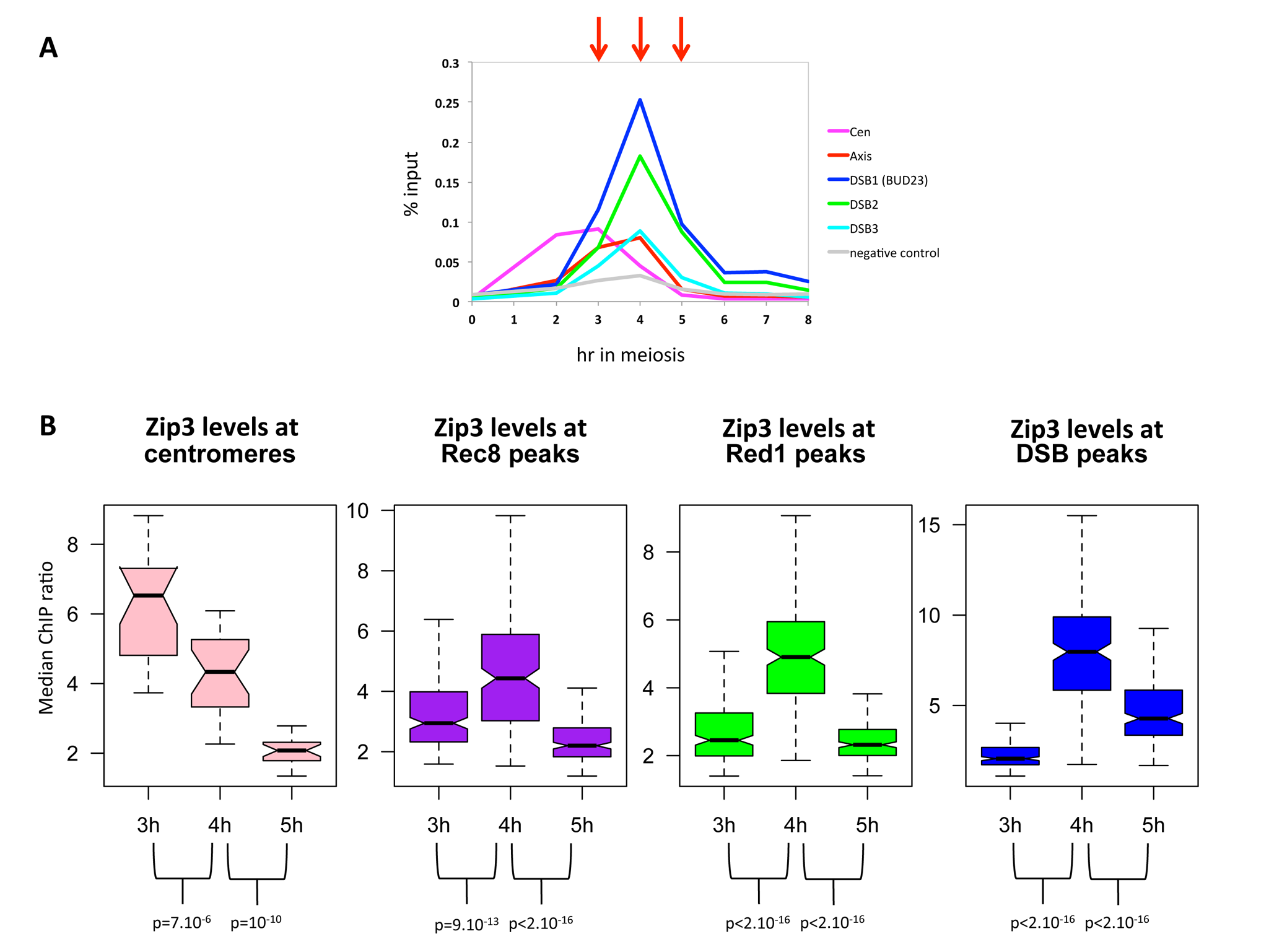

Supplement: Figure S1 — Genome-wide ChIPchip analysis of Zip3 at 3, 4 and 5 h in meiosis. (A) qPCR analysis of the Zip3-Flag ChIP samples used for ChIP-chip analysis. Zip3 association was monitored at the indicated regions in a wild-type strain (ORD9670). The average values from two independent time-courses are shown. The three red arrows indicate the time-points that were used in our ChIPchip analysis. (B) Global temporal variation of Zip3 association with centromeres, axis-association sites and DSBs. For each category, the following regions were considered: centromeres (Zip3 signal at probes at less than 200 bp from a centromere), Rec8, Red1 and DSBs (Zip3 signal at the 200 strongest Rec8, Red1 and DSB peaks, respectively). The decile-normalized ratios after denoising and smoothing using a 2 kb window are indicated. Boxplots show the median (line), 25th–75th percentile (box) ±1.5 times the interquartile range (whiskers). p value indicates the result of a Wilcoxon test between the two indicated time-points. (TIF) [file pgen.1003416.s001.tif]

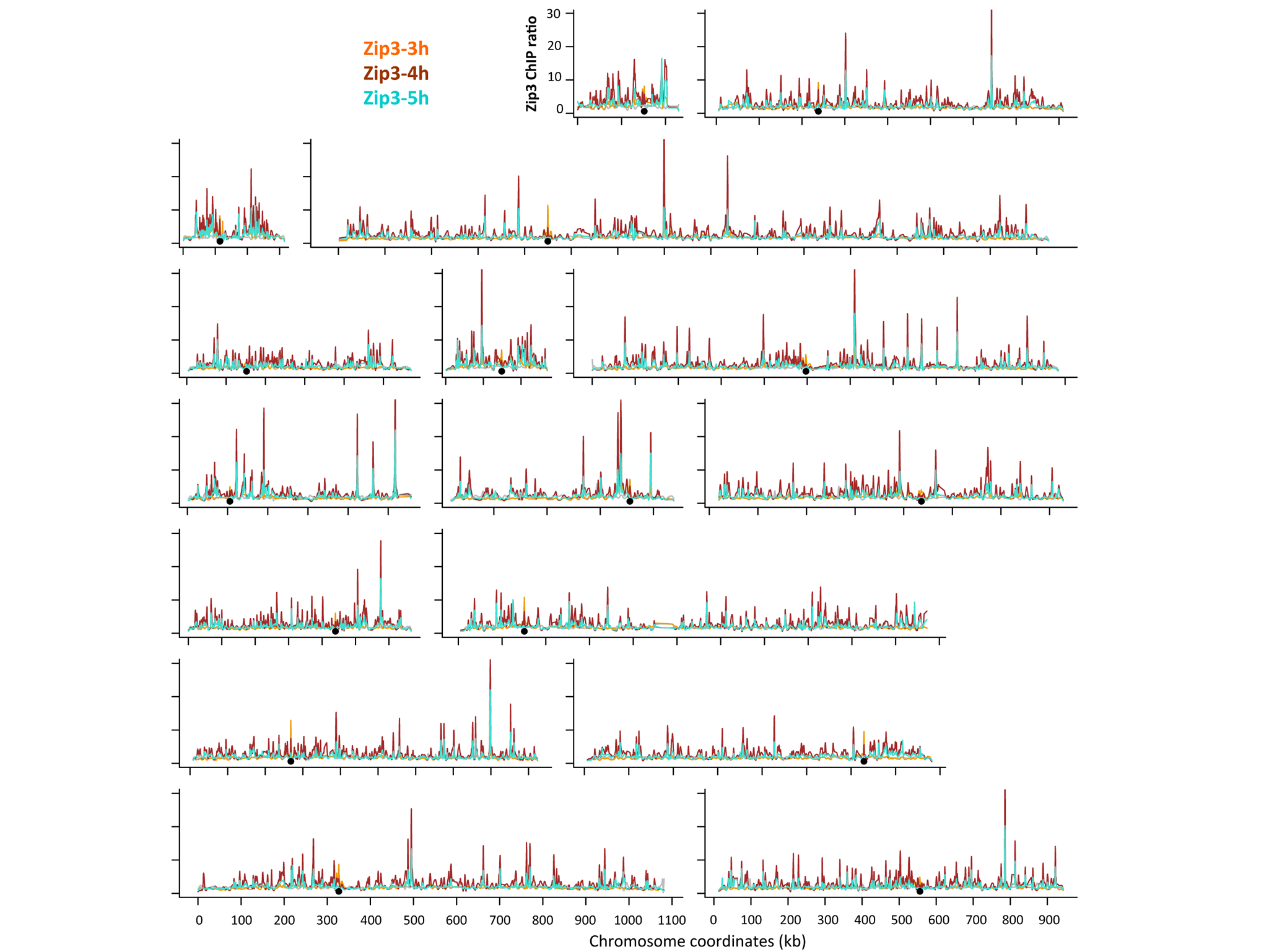

Supplement: Figure S2 — Genome-wide profiles of Zip3 localization. Average ChIP-chip Zip3-Flag decile-normalized ratios from two independent wild-type (ORD9670) meiotic time-courses are plotted after denoising and smoothing with a 1 kb window along the 16 chromosomes. Black circles indicate the centromere. Same experiment as in Figure S1. (TIF) [file pgen.1003416.s002.tif]

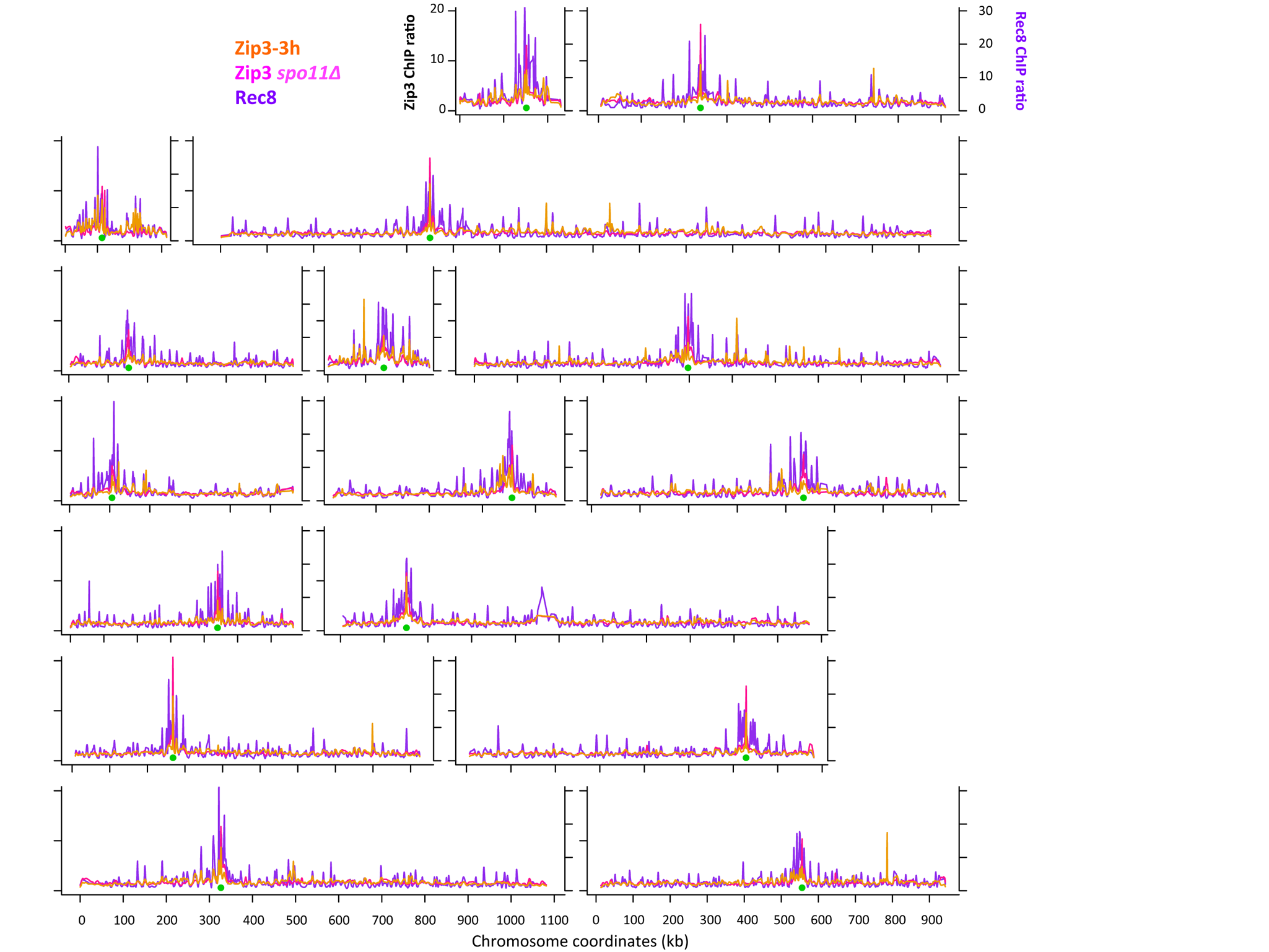

Supplement: Figure S3 — Genome-wide profiles of Zip3 ChIP at 3 hr in meiosis, Zip3 in a spo11Δ mutant and Rec8 Flag. Average decile-normalized ratios are plotted along the 16 chromosomes after denoising and 1 kb window smoothing. Green circles indicate the centromere. Rec8 data are from [23]. Zip3-Flag at 3 hr like in Figure S2 and spo11Δ at 3 hr is from ORD9684 strain. (TIF) [file pgen.1003416.s003.tif]

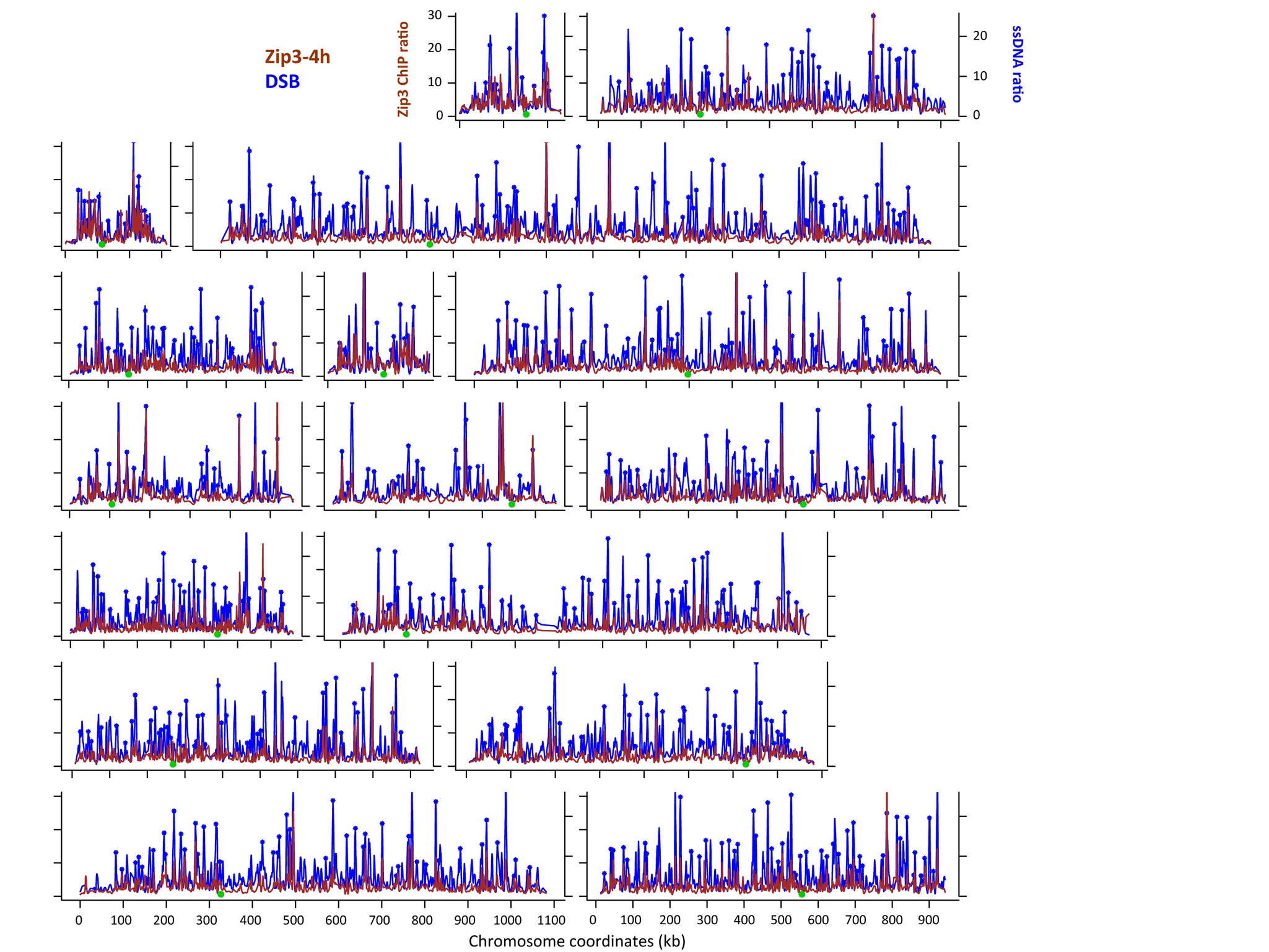

Supplement: Figure S4 — Genome-wide profiles of Zip3 ChIP at 4 hr and ssDNA accumulated at DSB ends in a dmc1Δ mutant (raw data from [3]). Average decile-normalized ratios are plotted along the 16 chromosomes after denoising and 1 kb window smoothing. Green circles indicate the centromere. Zip3-Flag at 4 hr like in Figure S2. Blue dots indicate DSB sites overlapping with a Zip3 peak. (TIF) [file pgen.1003416.s004.tif]

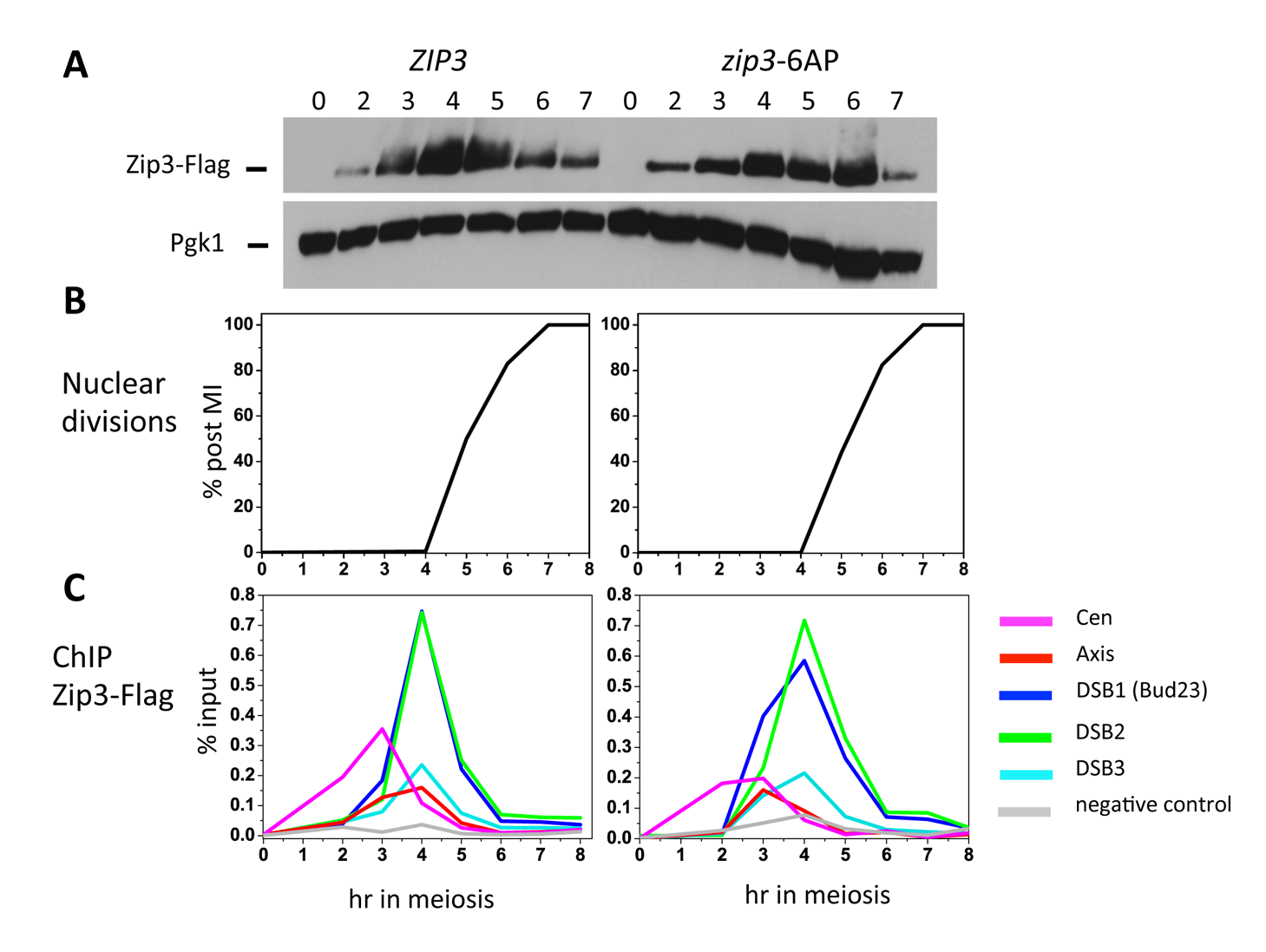

Supplement: Figure S5 — Mutation of the Zip3 consensus phosphorylation sites for the CDK kinase has no effect on Zip3 association with DSB sites. (A) Zip3-Flag expression in a wild-type (ORD9670) and zip3-6AP mutant strain (VBD1093) during a meiotic time-course. Zip3-Flag was monitored by western blotting with an anti-Flag antibody. Pgk1 served as loading control. (B) Meiotic progression in the same time-courses as in (A). Nuclear divisions were monitored by DAPI staining. (C) Monitoring of Zip3 binding in the same time-courses as in (A) and (B) by ChIP with an anti-Flag antibody and revealed by qPCR using primer pairs that cover the indicated regions. (TIF) [file pgen.1003416.s005.tif]

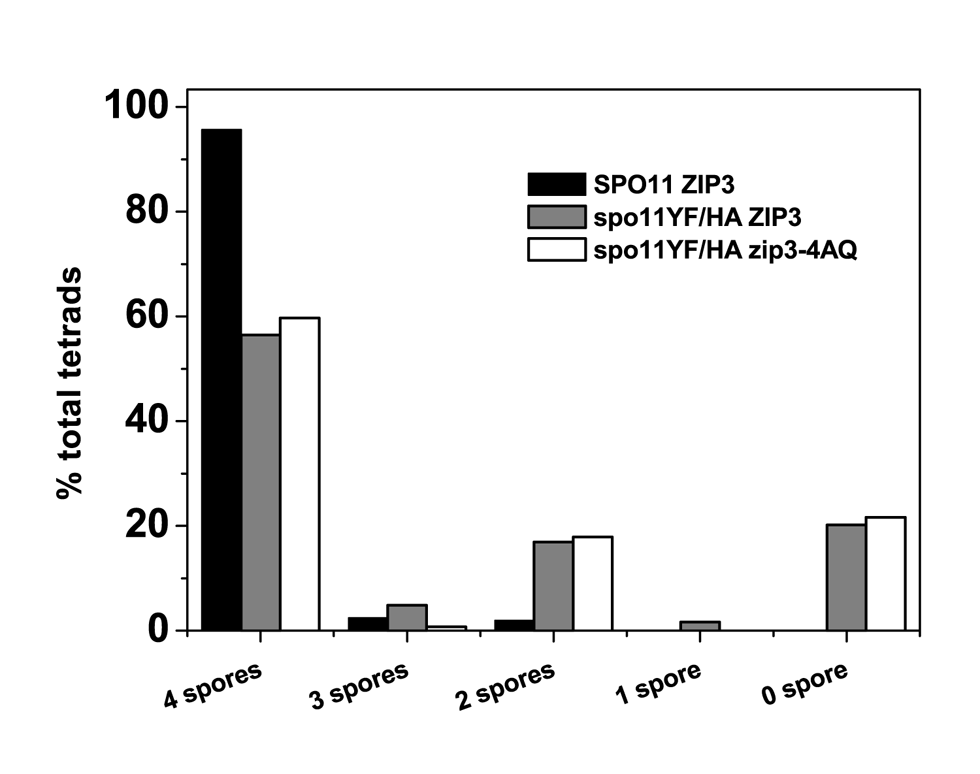

Supplement: Figure S6 — Spore viability in strains with reduced DSB formation and wild-type Zip3-Flag or mutant Zip34AQ-Flag. The proportion of 4, 3, 2, 1 or 0 viable spore per tetrad is indicated for each strain. SPO11 ZIP3: ORD9670 (205 tetrads); spo11YF/HA ZIP3: VBD1191 (124 tetrads); spo11YF/HA zip3-4AQ: VBD1192 (134 tetrads). (TIF) [file pgen.1003416.s006.tif]

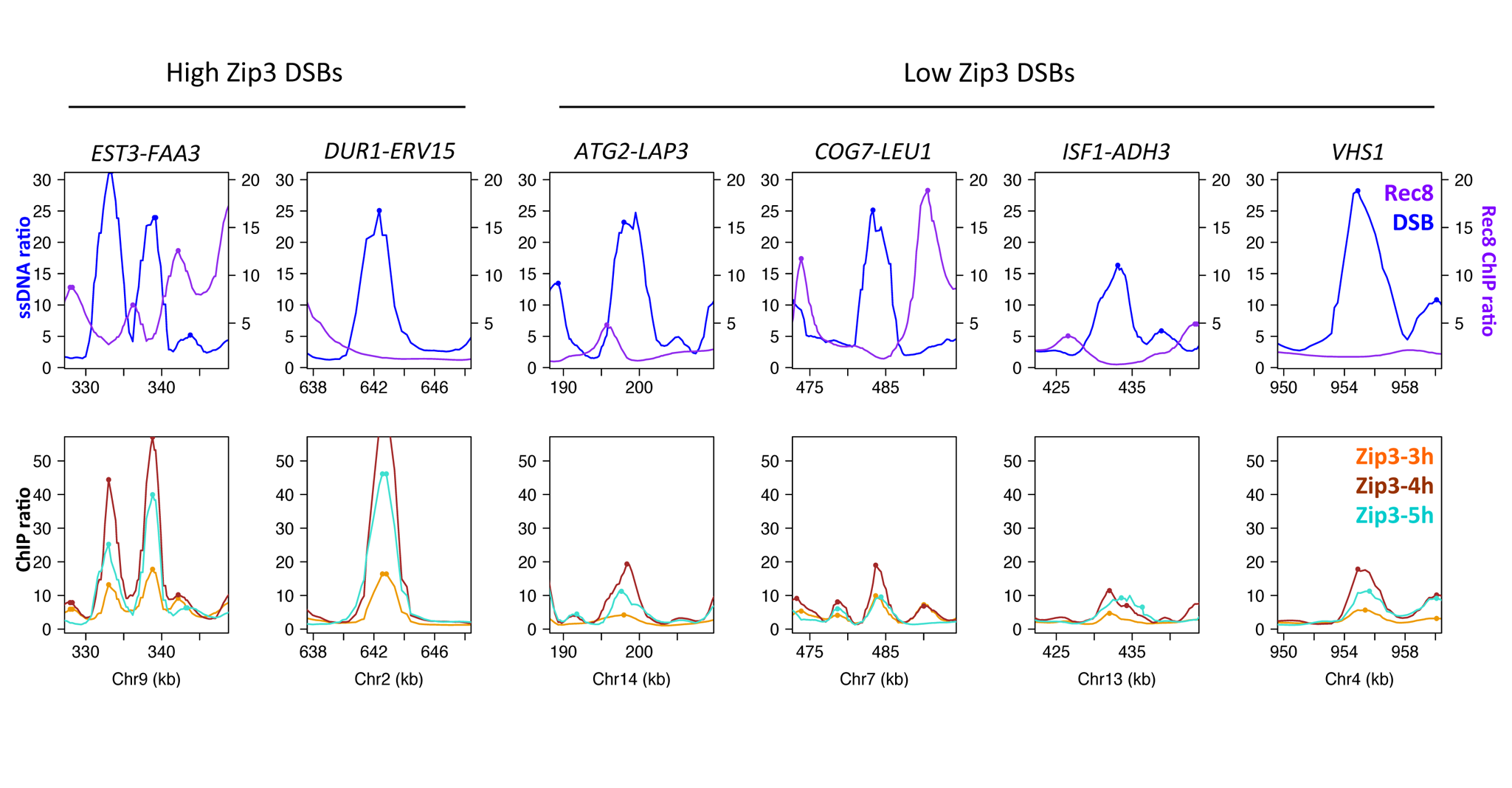

Supplement: Figure S7 — ChIP-chip profiles for Rec8, ssDNA and Zip3 around high- and low-Zip3 DSB sites. The actual site is at the center of each plot×axis. Decile-normalized ratios are represented, after denoising and smoothing with a 2 kb window. Dots indicate sites were a peak was detected. Same strains and experiments as in Figure 2. (TIF) [file pgen.1003416.s007.tif]

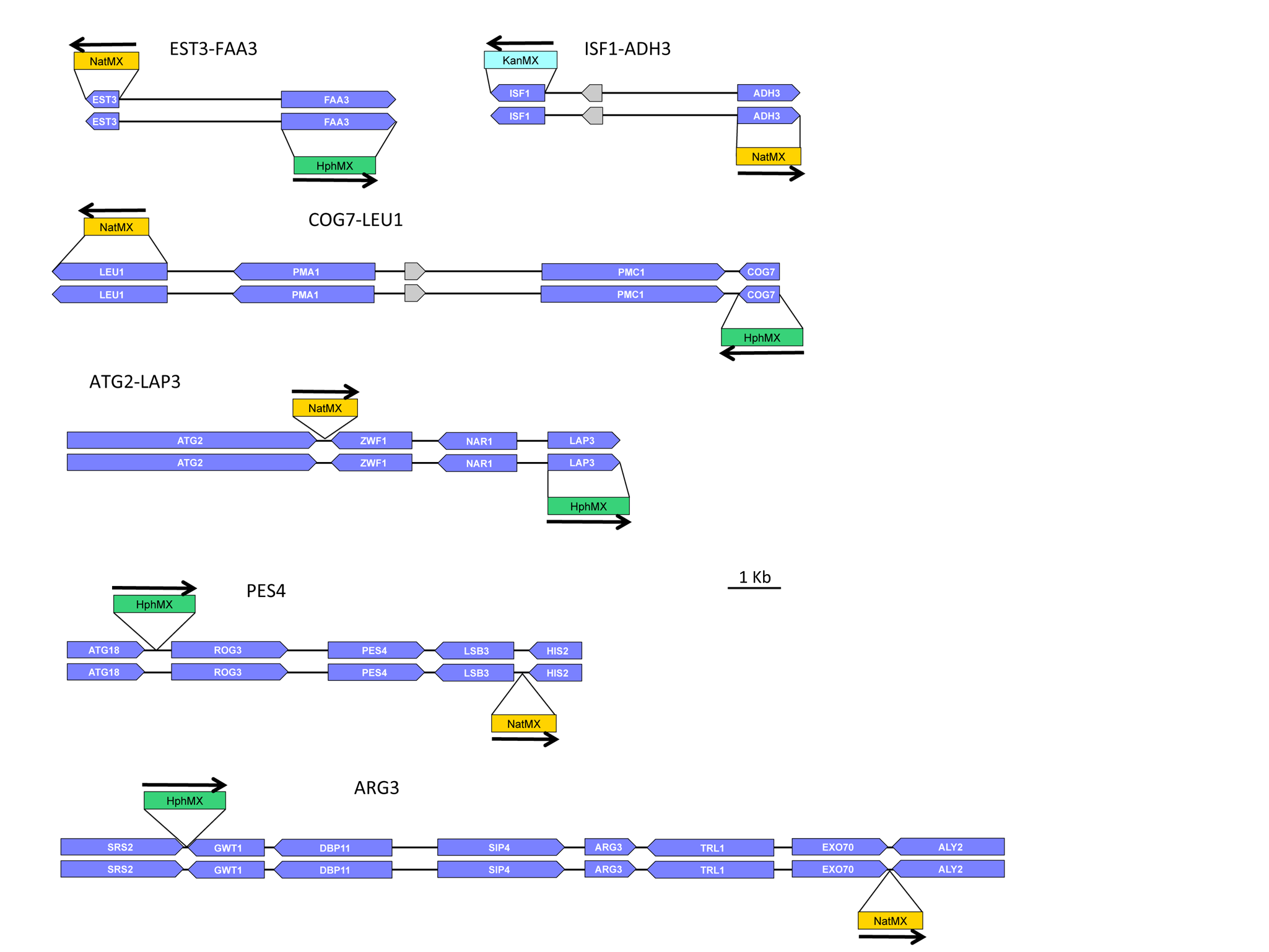

Supplement: Figure S8 — Schematic representation of the hemizygous flanking marker configuration used to assess genetic distances. (TIF) [file pgen.1003416.s008.tif]

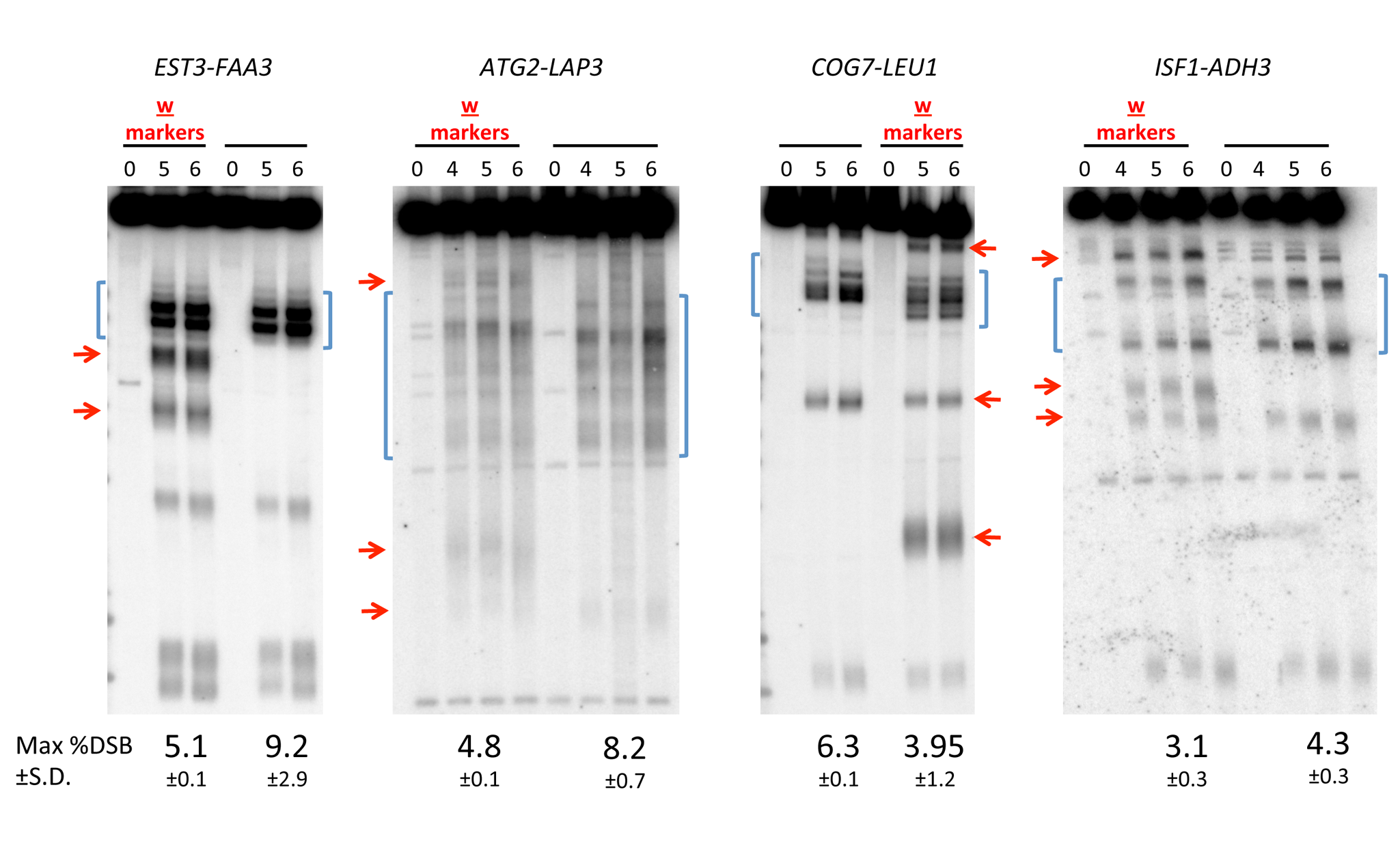

Supplement: Figure S9 — DSB frequencies in the chosen high-Zip3 and low-Zip3 intervals in the absence or presence of hemizygous flanking markers. Genomic DNA was extracted at the indicated time during meiosis from dmc1Δ cells and analyzed by Southern blotting. The brackets on the side of each panel indicate the physical interval comprised between the genetic recombination markers used to measure genetic distances. Red arrows indicate new DSB due to the insertion of a flanking marker. Below each panel is indicated the DSB frequency measured from at least two independent time-courses ± standard deviation. EST3-FAA3: with flanking markers: strain VBD1168; no markers: VBD1172. ATG2-LAP3: with flanking markers: strain VBD1218; no markers: VBD1172. COG7-LEU1: with flanking markers: strain VBD1172; no flanking markers: VBD1168. ISF1-ADH3: with flanking markers: strain VBD1170; no flanking markers: VBD1172. (TIF) [file pgen.1003416.s009.tif]

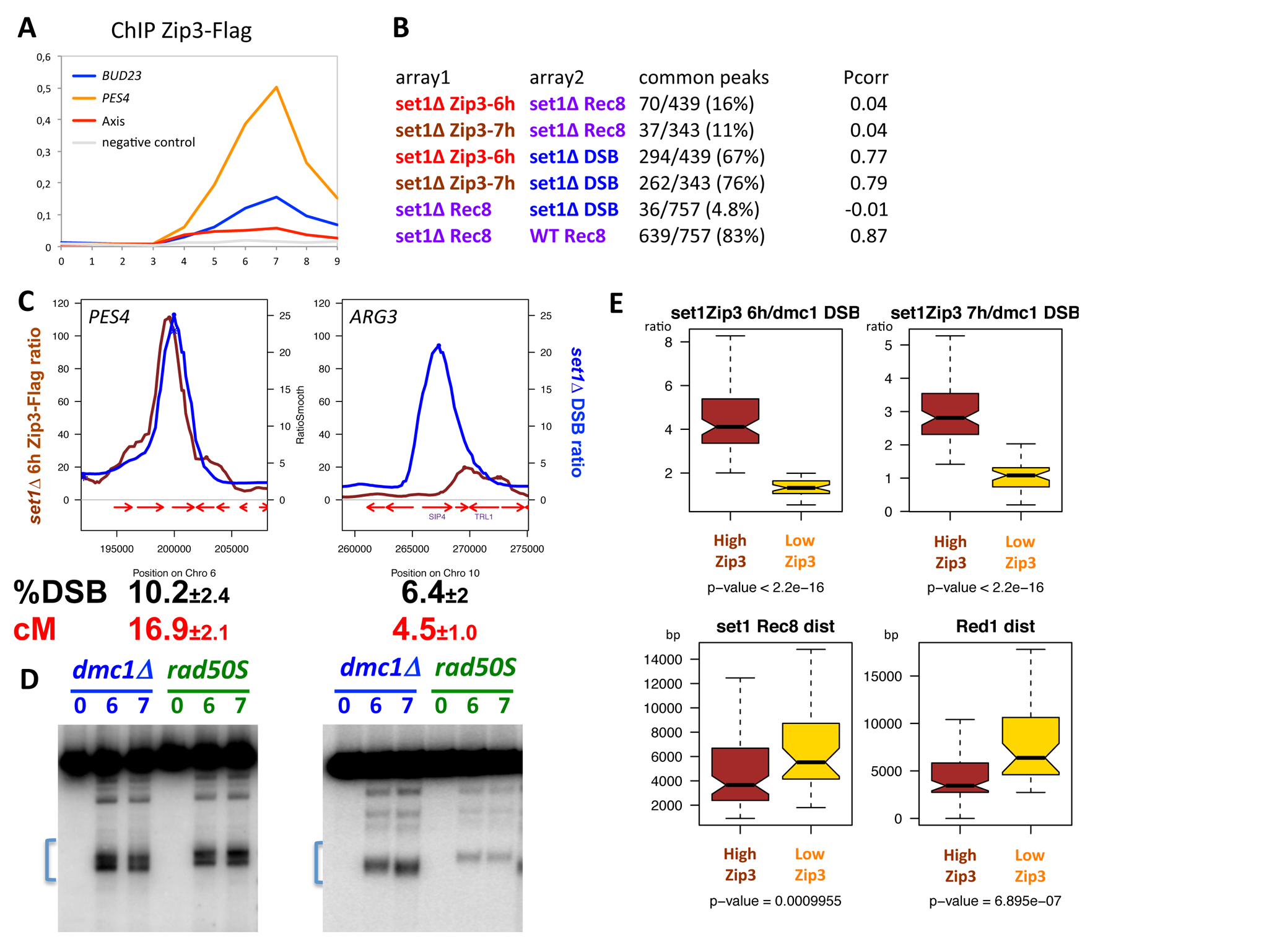

Supplement: Figure S10 — Zip3 associates with DSB hotspots in set1Δ with varying frequencies. (A) ChIP monitoring of Zip3-Flag association with the indicated regions during a meiotic time-course in set1Δ cells (VBD1005). (B) Comparison of the profiles between pairs of experiments. The name of each experiment is indicated, as well as the number of peaks in common between the two experiments and as percentage of the peaks of the first experiment. Pcorr assesses the linear Pearson's correlation coefficient between the profiles of the two experiments after denoising and smoothing with a 2 kb window. set1Δ DSB: raw data are from [33]; set1Δ Rec8: data are from [23]. (C) Comparison of Zip3-Flag binding and dmc1Δ DSBs in set1Δ strains in the PES4 and ARG3 regions, two sites with increased DSB frequency in the set1Δ mutant. set1Δ Zip3-Flag data are from the same time-course as in (A). set1Δ DSB raw data are from the Rpa ChIP-chip at 7 hr in a set1Δ dmc1Δ strain [33]. DSB frequencies were measured in dmc1Δ strains at 7 hr in meiosis (ORD9624) and values are from eight (PES4) and six (ARG3) independent experiments. Genetic distances were determined by scoring the segregation of hemizygous resistance markers flanking each interval (see Table S2 and details of the intervals in Figure S8). (D) Comparison of set1Δ DSB frequencies in the dmc1Δ (ORD9624) and rad50S (VBD1117) backgrounds at PES4 and ARG3 sites. DSB formation was measured by Southern blotting as described in Materials and Methods. Brackets indicate the interval in which genetic distances were measured. (E) Boxplot representation of the analysis of the indicated features at “High-Zip3” or “Low-Zip3” DSB sites (see details in the text). High-Zip3 DSB sites (n = 81) were selected among the strongest 200 set1Δ DSBs based on the presence of an associated Zip3 peak at 6 hr the signal intensity of which was less than 50 ranks away from that of the DSB site. Low-Zip3 DSB sites (n = 39) were selected among the strongest 200 set1Δ DSBs based on the ab [file pgen.1003416.s010.tif]

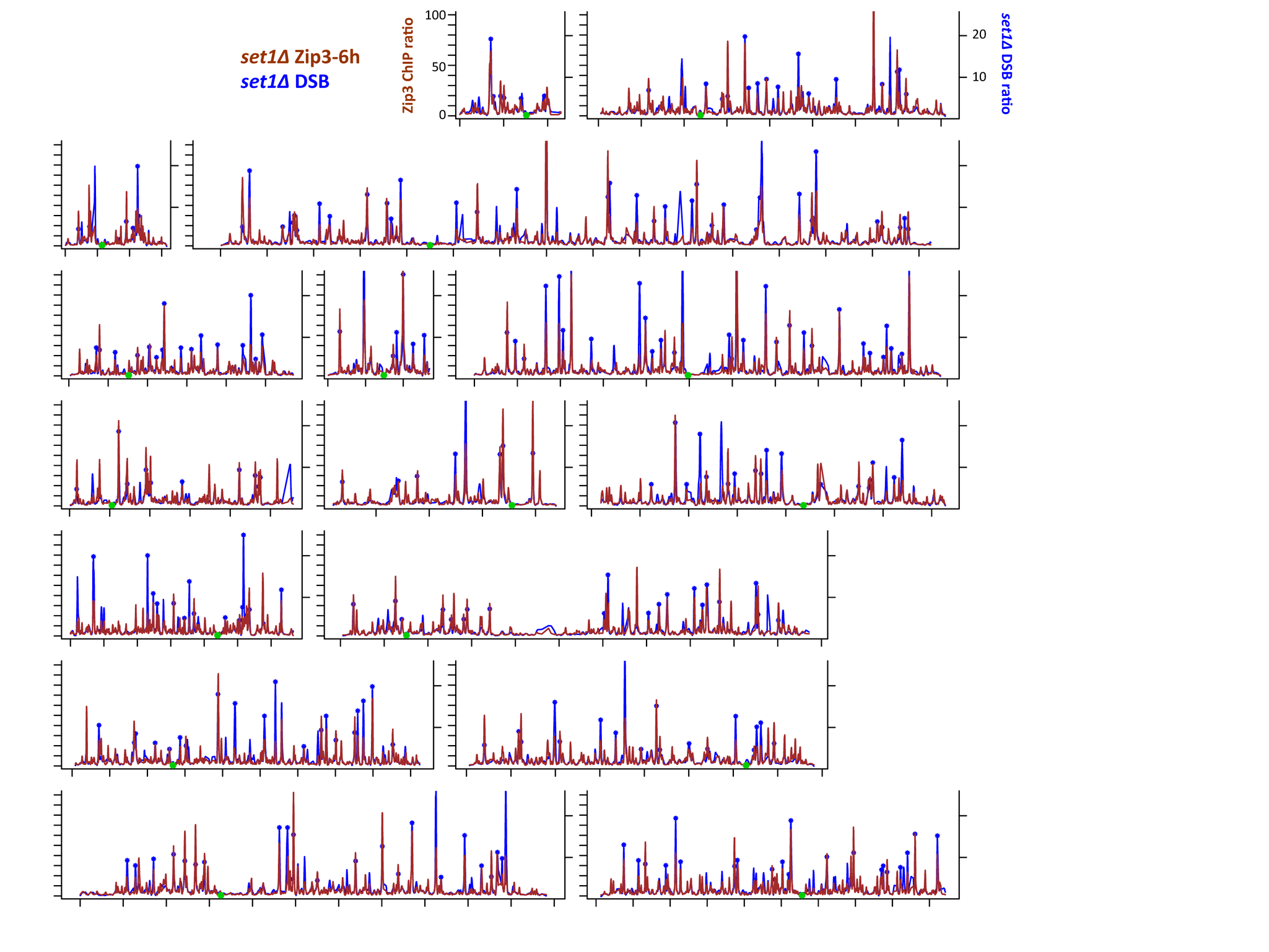

Supplement: Figure S11 — Genome-wide profiles of set1Δ ChIP-chip of Zip3 at 6 hr and RPA accumulated at DSB ends in a set1Δ dmc1Δ mutant (raw data from [33]). Decile-normalized ratios are plotted along the 16 chromosomes after denoising and 2 kb-window smoothing. Green circles indicate the centromere. Same experiment as in Figure S10C, with blue dots indicating DSB sites overlapping with a Zip3 peak. (TIF) [file pgen.1003416.s011.tif]

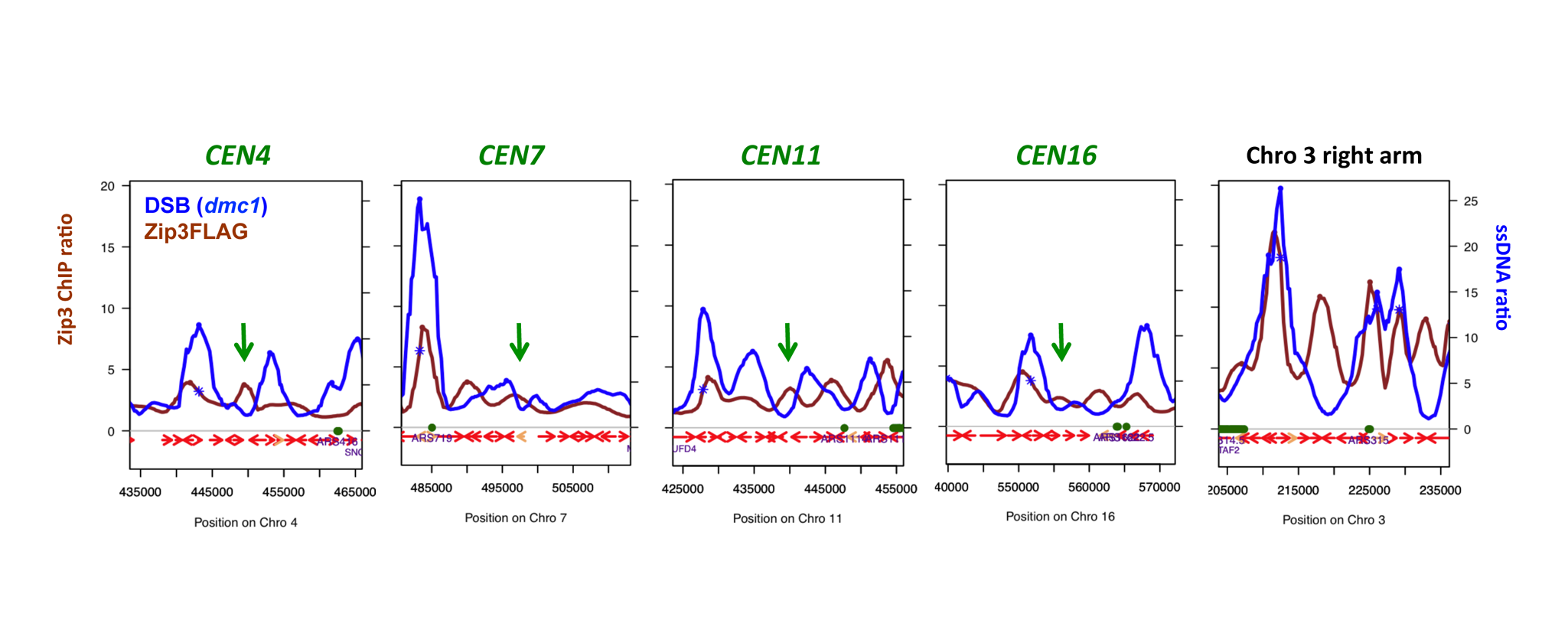

Supplement: Figure S12 — Association of Zip3 with centromere-proximal DSBs. Examples of Zip3 and DSB signals at four centromere regions and one chromosome arm. Graphs represent decile-normalized data after denoising and smoothing with a 2 kb window of Zip3 ChIP-chip at 4 hr or ssDNA at DSB. Same data as in Figure 6A. (TIF) [file pgen.1003416.s012.tif]

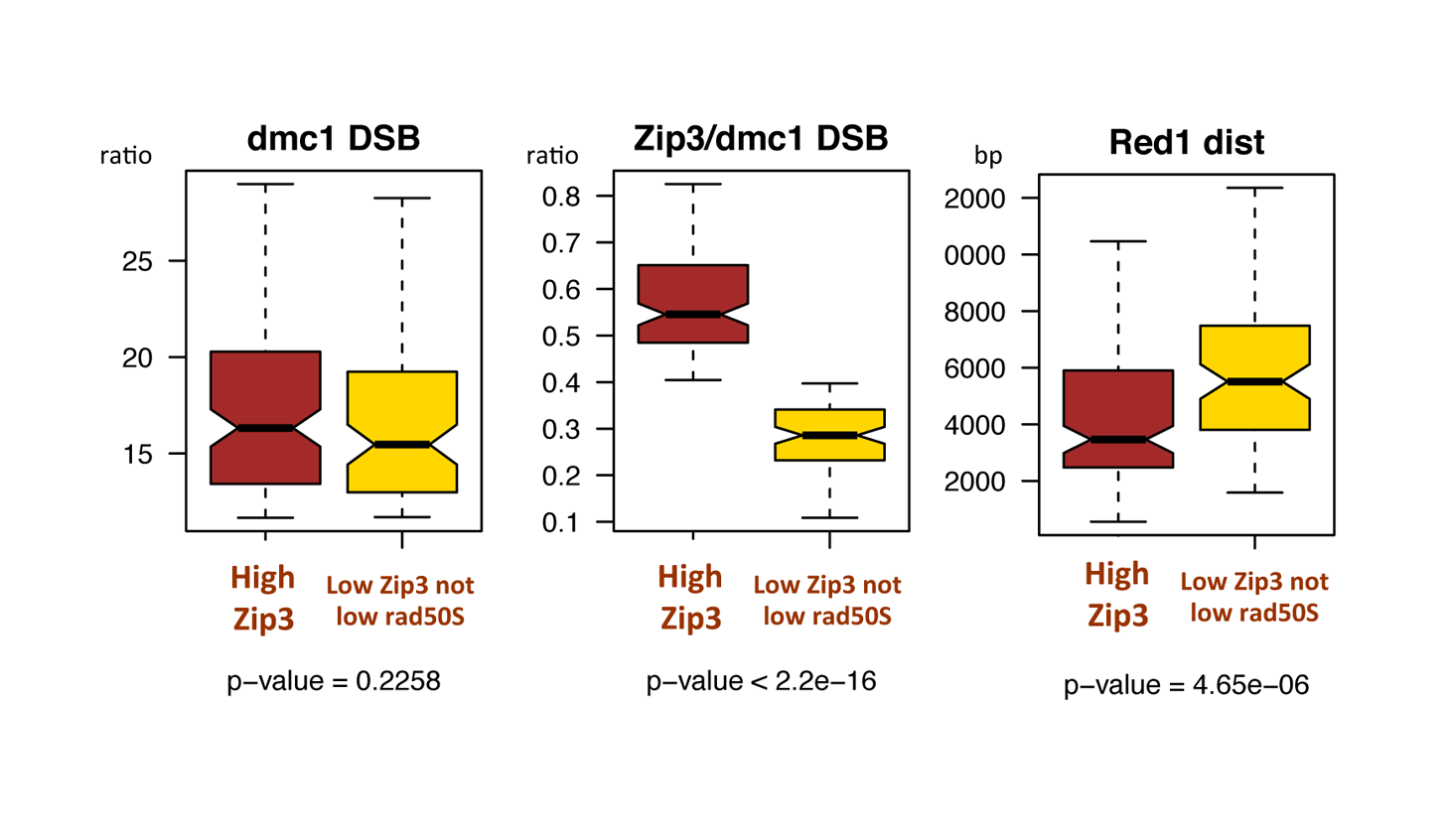

Supplement: Figure S13 — Features of the low-Zip3 DSB sites that are not low-rad50S DSBs (see details in the text). The rad50S and dmc1Δ DSB datasets are from [3]. Red1 binding data are from [24]. (TIF) [file pgen.1003416.s013.tif]
