# Supplementary material for: Differential Association of the Conserved SUMO Ligase Zip3 with Meiotic Double-Strand Break Sites Reveals Regional Variations in the Outcome of Meiotic Recombination
Source: PLoS Genet. 2013 Apr 4;9(4):e1003416. doi: 10.1371/journal.pgen.1003416 (PMC3616913; doi:10.1371/journal.pgen.1003416)
Supplement: Protocol S1 — Contains details about yeast strains construction, enzymes and probes used for DSB mapping and position of qPCR primers. (DOC) [file pgen.1003416.s014.doc]

**Protocol S1**

**Strain construction**

The His6-FLAG3 epitope was fused to the C-terminus of Zip3 by integrating a His6 –FLAG3–KanMX cassette amplified by PCR from the pU6H3FLAG plasmid (a gift from Kunihiro Otha) with flanking homology to the *ZIP3* endogenous locus.

The drug-resistance KanMX4, HphMX4 and NatMX4 cassettes were PCR-amplified from the plasmids pFA6a, pAG25 and pAG32 , respectively, with primers that have 50 bp flanking homology to the targeted locus, and integrated by transformation for precise replacement of the coding sequence of the following genes with the indicated resistance marker: *MND1*, *NDT80*, *PPH3*, *RAD52*, *ZIP1* (for the mutant analysis) and *EST3*, *FAA3*, *LAP3*, *ISF1*, *ADH3*, *COG7*, *LEU1* (for heterozygous flanking markers introduction around DSB sites). For flanking markers around the *PES4* and *ARG3* sites, the indicated drug resistance cassette was inserted with the same approach at nt 196400 of chromosome VI (*ATG18*-HphMX-*ROG3*), at nt 203700 of chromosome VI (*LSB3*-NatMX-*HIS2*), at nt 261000 of chromosome X (*SRS2*-HphMX-*GWT1*) and at nt 274780 of chromosome X (*EXO70*-NatMX-*ALY2*). For the flanking marker on the left of *ZWF1-LAP3* DSB, NatMX was inserted at nt 196264 of chromosome XIV (*ATG2*-NatMX-*ZWF1*).

**DSB and CO mapping**

DSBs at *BUD23*, *ARG3* and *PES4* were measured as described . For the other sites, restriction enzymes for genomic DNA digestion and coordinates of probes amplified by PCR were as follows: for *EST3-FAA3* DSB, *Bsp*EI and probe from nt 332422 to 331403 of chr IX; for *ATG2-LAP3* DSB, *Bam*HI and probe from nt 203874 to 204855 of chr XIV; for *ISF1-ADH3* DSB, *Bsp*EI and probe from nt 426703 to 429622 of chr XIII; for *COG7-LEU1* DSB, *Bam*HI and probe from nt 490971 to 491938 of chr VII. We noticed the presence of a Ty1 sequence (5920 bp) in the newly assembled SK1 genome (M. van Overbeek and S. Keeney, unpublished: <http://cbio.mskcc.org/public/SK1_MvO/>) between the *EST3* and *FAA3* genes that is not present in the S288C reference genome. For DSB frequency in flanking marker-containing strains, the following restriction enzymes and probe coordinates were used: for *EST3-FAA3* DSB, *Bsu36*I and probe *YIL006W* (nt 344062 to 345183 of chr IX); for *ATG2-LAP3* DSB, *Avr*II and probe *KEX2* (nt 202428 to 204420 of chr XIV); for *ISF1-ADH3* DSB, *Avr*II and probe part of *SEG1* (nt 439208 to 441090 of chr XIII); for *COG7-LEU1* DSB, *Ahd*I and probe part of *ERG4* (nt 473246 to 474276 of chr VII). CO frequency between the *est∆*::NatMX and *faa3∆*::HphMX drug resistance cassettes was measured after genomic DNA digestion with *Bsp*EI and *Bss*HII and PCR amplification of a probe from 337804bp to 338917 of chr IX.

**qPCR primers**

Primers amplified the following fragments: DSB1 (*BUD23*), chr III nt 211038 to 211152; DSB2, chr XV nt 495150 to 495213; DSB3, chr VI nt 74425 to 75177; Axis, chr III nt 232942 to 233010; Negative Control, chr III nt 279988 to 280050; CEN, chr XIII nt 267830 to 267931; PES4, chr VI nt 199403 to 199521.

**References**

1. Goldstein AL, McCusker JH (1999) Three new dominant drug resistance cassettes for gene disruption in Saccharomyces cerevisiae. Yeast 15: 1541-1553.

2. Wach A, Brachat A, Pohlmann R, Philippsen P (1994) New heterologous modules for classical or PCR-based gene disruptions in Saccharomyces cerevisiae. Yeast 10: 1793-1808.

3. Sommermeyer V, Beneut C, Chaplais E, Serrentino ME, Borde V (2013) Spp1, a Member of the Set1 Complex, Promotes Meiotic DSB Formation in Promoters by Tethering Histone H3K4 Methylation Sites to Chromosome Axes. Molecular Cell 49: 43-54.
