# Supplementary material for: Differential Association of the Conserved SUMO Ligase Zip3 with Meiotic Double-Strand Break Sites Reveals Regional Variations in the Outcome of Meiotic Recombination
Source: PLoS Genet. 2013 Apr 4;9(4):e1003416. doi: 10.1371/journal.pgen.1003416 (PMC3616913; doi:10.1371/journal.pgen.1003416)
Supplement: Table S4 — Strains used in this study. (DOC) [file pgen.1003416.s018.doc]

ORD7339 *a/l arg4-nsp,bgl/” leu2-R/leu2-K*

ORD9670 *a/l arg4-nsp,bgl/” leu2-R/leu2-K ZIP3-His6-Flag3::KanMX/”*

VBD1072 *a/l arg4-nsp,bgl/” leu2-R/leu2-K zip3H80A-His6-Flag3::KanMX/”*

VBD1073 *a/l arg4-nsp,bgl/” leu2-R/leu2-K zip3I96K-His6-Flag3::KanMX/”*

ORD9684 *a/l arg4-nsp,bgl/” leu2/”spo11∆::URA3/” ZIP3-His6-Flag3::KanMX/”*

ORD9688 *a/l arg4 leu2/”rad50S::LEU2/” ZIP3-His6-Flag3::KanMX/”*

ORD9699 *a/l arg4/” leu2-K/leu2-K dmc1∆::LEU2/” ZIP3-His6-Flag3::KanMX/”*

VBD1087 *a/l arg4-nsp,bgl/“ leu2-R/leu2-K mnd1∆::NatMX/” ZIP3-His6-Flag3::KanMX/”*

VBD1108 *a/l arg4-nsp,bgl/“ leu2-R/leu2-K rad52∆::NatMX/” ZIP3-His6-Flag3::KanMX/”*

VBD1001 *a/l arg4-nsp,bgl/” leu2/”ndt80∆::KanMX/” ZIP3- His6-Flag3::KanMX/”*

ORD9689 *a/l arg4-nsp,bgl/“ leu2-R/leu2-K zip1∆::KanMX/” ZIP3-His6-Flag3::KanMX/”*

VBD1093 *a/l arg4-nsp,bgl/“ leu2-R/leu2-K zip3-6AP-His6-Flag3::KanMX/”*

VBD1094 *a/l arg4-nsp,bgl/“ leu2-R/leu2-K zip3-4AQ-His6-Flag3::KanMX/”*

VBD1255 *a/l arg4-nsp,bgl/” leu2-R/leu2-K ZIP3-His6-Flag3::KanMX/” pph3∆::HphMX/”*

VBD1254 *a/l arg4-nsp,bgl/” leu2-R/leu2-K zip3-4AQ-His6-Flag3::KanMX/” pph3∆::HphMX/”*

VBH334/333 *a/l ho::hisG/” ade2/” ura3(∆Sma-Pst)/” his4-B/HIS4 LEU2/leu2-R CEN3/CEN3::ADE2 lys5-P/LYS5 MET13/ met13-B CYH2/cyh2-R TRP5/trp5-S CEN8::URA3/CEN8 ARG4/arg4-Bgl THR1/thr1-A cup1-S/CUP1 ZIP3-His6-Flag3::KanMX/”*

VBH332/331 *a/l ho::hisG/” ade2/” ura3(∆Sma-Pst)/” his4-B/HIS4 LEU2/leu2-R CEN3/CEN3::ADE2 lys5-P/LYS5 MET13/ met13-B CYH2/cyh2-R TRP5/trp5-S CEN8::URA3/CEN8 ARG4/arg4-Bgl THR1/thr1-A cup1-S/CUP1 zip3-4AQ-His6-Flag3::KanMX/”*

VBD1191 *a/l arg4-nsp,bgl/“ leu2/leu2 ZIP3-His6-Flag3 ::KanMX/” spo11-HA3His6::KanMX/spo11(Y135F)-HA3His6::KanMX*

VBD1192 *a/l arg4-nsp,bgl/“ leu2/leu2 zip3-4AQ-His6-Flag3 ::KanMX/” spo11-HA3His6::KanMX/spo11(Y135F)-HA3His6::KanMX*

VBD1022 *a/l arg4-nsp,bgl/“ leu2-R/leu2-K FAA3/faa3∆::HphMX est3∆::NatMX/EST3*

VBD1229 *a/l arg4-nsp,bgl/“ leu2-R/leu2-K FAA3/faa3∆::HphMX est3∆::NatMX/EST3 ZIP3-His6-Flag3 ::KanMX/”*

VBD1113 *a/l arg4-nsp,bgl/“ leu2-R/leu2-K FAA3/faa3∆::HphMX est3∆::NatMX/EST3 zip3-4AQ-His6-Flag3::KanMX/”*

VBD1244 *a/l arg4-nsp,bgl/“ leu2-R/leu2-K FAA3/faa3∆::HphMX est3∆::NatMX/EST3 ZIP3-His6-Flag3 ::KanMX/” mus81∆::KanMX/”*

VBD1245 *a/l arg4-nsp,bgl/“ leu2-R/leu2-K FAA3/faa3∆::HphMX est3∆::NatMX/EST3 zip3-4AQ-His6-Flag3::KanMX/” mus81∆::KanMX/”*

VBD1205 *a/l arg4-nsp,bgl/“ leu2-R/leu2-K ATG2-ZWF1/ATG2-NatMX-ZWF1* *lap3∆::HphMX/LAP3*

VBD1064 *a/l arg4-nsp,bgl/“ leu2-R/leu2-K ISF1/isf1∆::KanMX adh3∆::NatMX/ADH3*

VBD1157 *a/l arg4-nsp,bgl/“ leu2-R/leu2-K cog7∆::HphMX/COG7 LEU1/leu1∆::NatMX*

VBD1168 *a/l arg4-nsp,bgl/“ leu2-R/leu2-K FAA3/faa3∆::HphMX est3∆::NatMX/EST3 dmc1∆::LEU2/”*

VBD1218 *a/l arg4-nsp,bgl/“ leu2-R/leu2-K ATG2-ZWF1/ATG2-NatMX-ZWF1* *lap3∆::HphMX/LAP3 dmc1∆::LEU2/”*

VBD1170 *a/l arg4-nsp,bgl/“ leu2-R/leu2-K ISF1/isf1∆::KanMX adh3∆::NatMX/ADH3 dmc1∆::LEU2/”*

VBD1172 *a/l arg4-nsp,bgl/“ leu2-R/leu2-K cog7∆::HphMX/COG7 LEU1/leu1∆::NatMX dmc1∆::LEU2/”*

VBD1005 *a/l arg4-nsp,bgl/” leu2/”set1∆::KanMX/” ZIP3- His6-Flag3::KanMX/”*

ORD9624 *a/l arg4/” leu2-K/leu2-K set1∆::KanMX/”dmc1∆::LEU2/”*

VBD1117 *a/l arg4/” leu2/” set1∆::KanMX/”rad50S::LEU2/”*

VBD1080 *a/l arg4-nsp,bgl/” leu2-R/leu2-K ATG18-ROG3/ATG18::HphMX::ROG3 LSB3-HIS2/LSB3::NatMX::HIS2 set1∆::KanMX/”*

VBD1081 *a/l arg4-nsp,bgl/” leu2-R/leu2-K SRS2-GWT1/SRS2::HphMX::GWT1 EXO70-ALY2/EXO70::NatMX::ALY2 set1∆::KanMX/”*

# Table S4: strains used in this study. All strains are of the SK1 background and are *ura3/” lys2/” ho::LYS2/”* unless otherwise stated. The a parent is indicated first.
